# Fcγ-Receptor-Based Enzyme-Linked Immunosorbent Assays for Sensitive, Specific, and Persistent Detection of Anti-SARS-CoV-2 Nucleocapsid Protein IgG Antibodies in Human Sera

**DOI:** 10.1128/jcm.00075-22

**Published:** 2022-05-16

**Authors:** Christina Deschermeier, Christa Ehmen, Ronald von Possel, Carolin Murawski, Ben Rushton, John Amuasi, Nimako Sarpong, Oumou Maiga-Ascofaré, Raphael Rakotozandrindrainy, Danny Asogun, Yemisi Ighodalo, Lisa Oestereich, Sophie Duraffour, Meike Pahlmann, Petra Emmerich

**Affiliations:** a Diagnostics Development Laboratory, Bernhard Nocht Institute for Tropical Medicine, Hamburg, Germany; b Department for Virology, Bernhard Nocht Institute for Tropical Medicine, Hamburg, Germany; c Department of Tropical Medicine and Infectious Diseases, Center of Internal Medicine II, University of Rostock, Rostock, Germany; d Global Health and Infectious Disease Research Group, Kumasi Centre for Collaborative Research in Tropical Medicine, Kumasi, Ghana; e Infectious Disease Epidemiology Research Group, Kumasi Centre for Collaborative Research in Tropical Medicine, Kumasi, Ghana; f German Center for Infection Research (DZIF), Hamburg-Lübeck-Borstel-Riems, Hamburg, Germany; g Department for Microbiology and Parasitology, University of Antananarivo, Antananarivo, Madagascar; h Institute of Lassa Fever Research and Control, Irrua Specialist Teaching Hospital, Irrua, Nigeria; Cepheid

**Keywords:** immunoassay, infectious disease, immunoglobulins, laboratory methods and tools, viral diseases

## Abstract

Sensitive and specific serological tests are mandatory for epidemiological studies evaluating severe acute respiratory syndrome coronavirus 2 (SARS-CoV-2) prevalence as well as coronavirus disease 2019 (COVID-19) morbidity and mortality rates. The accuracy of results is challenged by antibody waning after convalescence and by cross-reactivity induced by previous infections with other pathogens. By employing a patented platform technology based on capturing antigen-antibody complexes with a solid-phase-bound Fcγ receptor (FcγR) and truncated nucleocapsid protein as the antigen, two SARS-CoV-2 IgG enzyme-linked immunosorbent assays (ELISAs), featuring different serum and antigen dilutions, were developed. Validation was performed using a serum panel comprising 213 longitudinal samples from 35 COVID-19 patients and a negative-control panel consisting of 790 pre-COVID-19 samples from different regions of the world. While both assays show similar diagnostic sensitivities in the early convalescent phase, ELISA 2 (featuring a higher serum concentration) enables SARS-CoV-2 IgG antibody detection for a significantly longer time postinfection (≥15 months). Correspondingly, analytical sensitivity referenced to indirect immunofluorescence testing (IIFT) is significantly higher for ELISA 2 in samples with a titer of ≤1:640; for high-titer samples, a prozone effect is observed for ELISA 2. The specificities of both ELISAs were excellent not only for pre-COVID-19 serum samples from Europe, Asia, and South America but also for several challenging African sample panels. The SARS-CoV-2 IgG FcγR ELISAs, methodically combining antigen-antibody binding in solution and isotype-specific detection of immune complexes, are valuable tools for seroprevalence studies requiring the (long-term) detection of anti-SARS-CoV-2 IgG antibodies in populations with a challenging immunological background and/or in which spike-protein-based vaccine programs have been rolled out.

## INTRODUCTION

Reliable serological assays are mandatory for monitoring the progression of the severe acute respiratory syndrome coronavirus 2 (SARS-CoV-2) pandemic, particularly in regions of the world where access to medical care and molecular testing capacities are limited. To enable a conclusive estimation of SARS-CoV-2 seroprevalence, a SARS-CoV-2 IgG assay has to fulfill several crucial requirements. First, due to postconvalescence antibody waning (1), high assay sensitivity in coronavirus disease 2019 (COVID-19) patient samples collected more than 6 months after infection is needed. Second, although SARS-CoV-2 IgG assay specificity in general is good to excellent in sample panels from Europe, Asia, and the United States (2), several African serum panels were recently shown to strongly challenge assay specificity due to a different immunological background caused by endemic infectious diseases such as Plasmodium falciparum malaria and others (3–6). Therefore, if use in these settings is intended, critical assessment and (where necessary) optimization of assay specificity are required. Third, with the progressive rollout of country-specific vaccine programs, humoral immune responses induced by natural SARS-CoV-2 infection must be differentiated from vaccine responses. If spike-based vaccines like, e.g., Comirnaty (BioNTech/Pfizer) or Vaxzevria (AstraZeneca) are administered, assays employing the SARS-CoV-2 nucleocapsid protein (NCP) (7) as the antigen still allow the unequivocal detection of antibodies induced by natural SARS-CoV-2 infection. Notably, highly sensitive assays will be required to detect the apparently reduced humoral anti-NCP response that has been observed in vaccine breakthrough infections (8). In contrast, vaccines based on inactivated SARS-CoV-2 (e.g., the Sinopharm vaccine) potentially elicit antibodies against all structural virus proteins. In principle, natural infection in individuals immunized with those vaccines could be proven by the detection of antibodies targeting nonstructural proteins like open reading frame 3 (ORF3) or ORF8 (9). Nevertheless, several SARS-CoV-2 lineages lacking the functional expression of these proteins have been described (10, 11).

During the last few years, we have employed a patented platform technology (12, 13) methodically combining antigen-antibody binding in solution with isotype-specific detection of *in vitro-*formed immune complexes to develop several highly sensitive and specific plate-based enzyme-linked immunosorbent assays (ELISAs) for the detection of both IgM and IgG antibodies directed against viral pathogens such as dengue virus (DENV) (14), Lassa virus (LASV) (15), Crimean-Congo hemorrhagic fever virus (CCHFV) (16), and Zika virus (ZIKV) (17, 18). Briefly, for the detection of IgG, patient sera are coincubated with soluble, labeled recombinant antigen (and, if necessary, unlabeled competitor molecules suppressing cross-reactive antibody binding) on a 96-well plate coated with the recombinantly produced immunoglobulin-like (Igl) domain of *Homo sapiens* Fcg receptor IIA_H131_ (*Hs*FcγRIIA_H131_) (mediating the isotype-specific binding of IgG-antigen immune complexes). Thereby, the use of natively folded antigen in the liquid phase guarantees the preservation of conformational epitopes and prevents nonspecific interactions with hydrophobic amino acid stretches that may be exposed in partially unfolded or denatured proteins. If necessary, even high serum concentrations can be applied without increasing the assay background (19). Captured immune complexes are then detected by a colorimetric reaction that can be quantified using a conventional ELISA reader.

Here, we utilize this technology to develop ELISA protocols for the detection of antibodies directed against the SARS-CoV-2 NCP and present detailed validation data on reproducibility, diagnostic and analytical sensitivities, specificity, and interference.

## MATERIALS AND METHODS

### Generation of prokaryotic expression vectors (pOPIN-J-CoV-N2b/SB/N3).

Amplicons encoding the N2b/SB/N3 domain (7) of coronavirus (CoV) NCPs were generated by PCR (see Table S1 in the supplemental material) using either random-primed cDNA transcribed from virus RNA or synthesized cDNA (codon optimized for expression in Escherichia coli; GenScript) as a template and inserted by in-fusion cloning (TaKaRa) into pOPIN-J (20) cut with HindIII/KpnI. Insert sequences were verified using Sanger sequencing (Seqlab).

### Recombinant expression and purification of recombinant antigens.

**FIG 1:**
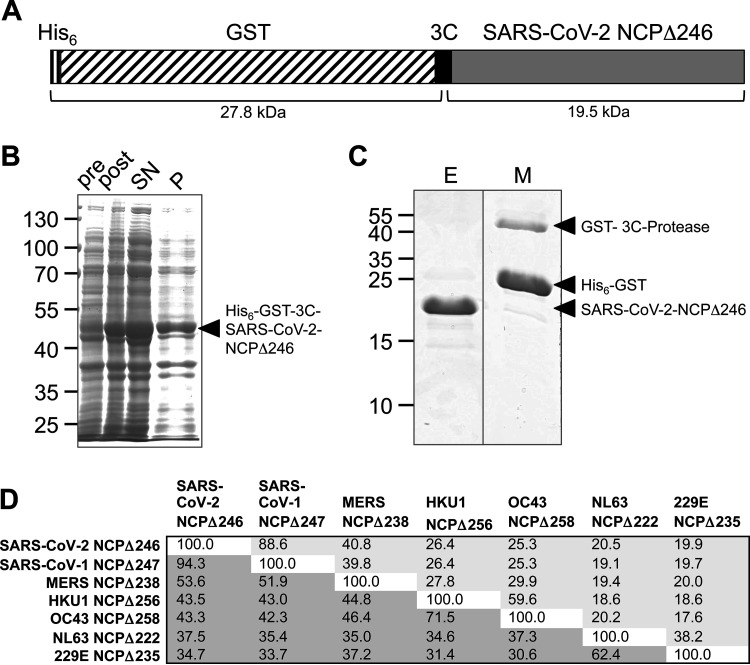
Prokaryotic expression and purification of N-terminally truncated SARS-CoV-2 NCP. (A) Schematic representation of the fusion protein His_6_–GST–3C–SARS-CoV-2 NCPΔ246 (calculated molecular weight, 47.3 kDa). (B) Total lysates preinduction (pre) and postinduction (post) and soluble (supernatant [SN]) and insoluble (pellet [P]) lysate fractions. (C) Eluate (E) and matrix (M) after on-column cleavage. (D) Amino acid sequence comparison of truncated CoV NCPs. Above the diagonal/light gray shading are identity scores (percent), and below the diagonal/dark gray shading are similarity scores (percent). MERS, Middle East respiratory syndrome.

Expression vectors encoding tandem-affinity-tagged, N-terminally truncated CoV NCPs (Fig. 1A) were transformed into E. coli BL21 RIG cells. Upon induction with 1 mM isopropyl-β-d-thiogalactopyranoside (IPTG), recombinant fusion proteins were expressed for 3 h at 30°C or 37°C. Harvested bacteria were lysed, and fusion proteins were purified from the soluble fraction of the bacterial lysate (Fig. 1B) by Ni-nitrilotriacetic acid (NTA) chromatography under native conditions followed by on-column cleavage of the tandem His_6_–glutathione *S*-transferase (GST) tag (Fig. 1C) as described previously (16).

### Biotinylation of recombinant antigens.

Purified, recombinant antigens were covalently coupled to biotin using EZ-Link sulfo-NHS (*N*-hydroxysuccinimide)-biotin reagent (Thermo Fisher) at a 30-fold molar excess according to the manufacturer’s instructions. Biotinylated proteins were stored in 0.5× phosphate-buffered saline (PBS)–50% glycerol at −20°C.

### Human sera.

This study complies with the Declaration of Helsinki. Written informed consent was obtained from all individuals or, in the case of minors, from parents or legal guardians before enrollment. Data privacy protection was guaranteed by the anonymization of samples.

### (i) COVID-19 patient sera.

Longitudinal serum samples (range of days after the onset of symptoms [days postonset {dpo}], 10 to 446; number of serum samples per patient, 1 to 13) from 35 patients (median age, 45 years; age range, 19 to 64 years; 23 females and 12 males) with PCR-confirmed SARS-CoV-2 infection collected in Germany between March 2020 and May 2021 were analyzed. All patients displayed ambulatory mild disease (21). Fifteen patients received a COVID-19 vaccination after more than 6 months of convalescence (10 BioNTech/Pfizer, 2 Moderna, and 3 AstraZeneca). The collection of samples was approved by the Medical Association, Hamburg, Germany (no. 2020-10162-BO-ff). World Health Organization (WHO) international standard plasma 20/136 (1,000 binding antibody units [BAU]/mL) was obtained from the National Institute for Biological Standards and Control (NIBSC).

### (ii) *A priori* SARS-CoV-2 IgG-negative control sera.

The study was performed using stored human serum/plasma samples from symptom-free donors collected before the COVID-19 pandemic in Germany (2004 to 2015), Ghana (1999 and 2014 to 2015), Madagascar (2010), Nigeria (2018), Colombia (2014), and Lao People’s Democratic Republic (PDR) (2014) (Table S2) (3). The collection of samples was approved by the Ethics Committees of the Kwame Nkrumah University of Science and Technology (Kumasi, Ghana) (CHRPE/AP/427/13, 2013), the Comité d’Ethique de la Vice Primature Chargée de la Santé Publique (Antananarivo, Madagascar) (no. 051-CE/MINSAN, 2009), the Irrua Specialist Teaching Hospital (Irrua, Nigeria) (ISTH/HREC/20171019/28, 2017), the Hospital Rosario Pumarejo de Lopez (Valledupar, Colombia) (2013), Lao People’s Democratic Republic (no. 030/NECHR), and the Medical Association, Hamburg, Germany (no. PV4608, 2013).

### SARS-CoV-2 IgG indirect immunofluorescence testing (IIFT).

Immunofluorescence analysis detecting IgG antibodies targeting SARS-CoV-2 full-virus antigens was performed as described previously (22). Briefly, SARS-CoV-2-infected Vero-E6 cells (ATCC CRL-1586) were fixed with acetone-methanol and incubated with serial dilutions of patient sera. After washing, the detection of bound IgG was performed using a fluorescein isothiocyanate (FITC)-labeled anti-IgG antibody (Sifin).

### SARS-CoV-2 IgG ELISAs 1 and 2.

For the SARS-CoV-2 IgG FcγR ELISA, Nunc MaxiSorp ELISA plates were coated with 5 μg/mL *Hs*FcγR-Igl (CD32 [FcγRIIA_H131_]) (14) in PBS (pH 7.4). After blocking for 2 h at room temperature with 1× PBS (pH 7.4)–0.25% bovine serum albumin (BSA)–0.05% Tween 20, plates were stabilized with a liquid plate sealer (Candor) and stored at 4°C until use. Human sera (ELISA 1, 1:50 in 1× PBS [pH 7.4]–0.05% ProClin 300–0.01% phenol red; ELISA 2, pure sera) and biotinylated recombinant antigen (ELISA 1, 1:10,000; ELISA 2, 1:25,000) (in conjugate dilution buffer [CDB] [1× PBS {pH 7.4}–1% BSA–0.5% fetal bovine serum–1% Nonidet P-40–0.1% ProClin 300]) were mixed 1:1 on the *Hs*FcγR-Igl-coated plates and coincubated overnight at 4°C. After washing with 100 mmol/L Tris-HCl (pH 7.4)–150 mmol/L NaCl–0.05% Tween 20–0.005% ProClin 300, horseradish peroxidase (HRP)-labeled streptavidin (0.1 μg/mL in CDB) was applied to the wells for 1 h at 4°C. After washing again, tetramethylbenzidine (TMB; KPL) was added for 20 min at room temperature, and the reaction was stopped by the addition of 1 N sulfuric acid (Merck). The TMB reaction product was quantified by measuring the absorbances at 450 nm (*A*450) and 620 nm (*A*620) on a SPECTROstar Nano ELISA reader (BMG Labtech) and calculating the difference *A*450-*A*620. The Euroimmun anti-SARS-CoV-2 NCP ELISA (IgG) was performed and evaluated according to the manufacturer’s instructions.

### Common cold CoV IgG ELISAs.

IgG antibodies interacting with the N-terminally truncated NCPs of OC43, HKU1, NL63, and 229E were detected in human sera as described previously (3). Briefly, IgG FcγR ELISA 1 described above was performed using biotinylated OC43 NCPΔ258, HKU NCPΔ256, NL63 NCPΔ222, and 229E NCPΔ258 (Table S1), respectively, as the antigens.

### Interference testing.

To evaluate interference by hemolytic, icteric, and lipemic serum conditions as well as high biotin and rheumatoid factor (RF) concentrations, assay mixtures were spiked with hemoglobin (Sigma), bilirubin (Sigma), triglycerides (Sigma), biotin (Sigma), and RF (Lee Biosolutions), respectively. Simulated serum concentrations were 5 mg/mL and 10 mg/mL for hemoglobin, 0.5 mg/mL for bilirubin, and 5 mg/mL for triglycerides. Biotin interference was tested at simulated serum concentrations of between 0.1 ng/mL and 100,000 ng/mL; simulated RF serum concentrations were 50 IU/mL, 100 IU/mL, and 1,000 IU/mL. Assays were performed by employing three COVID-19 patient serum samples covering a range of *A*_450_-*A*_620_ readings (“low,” “medium,” and “high”) and at least three prepandemic negative-control serum samples.

### Sequence alignments and protein structure visualization.

Multiple-amino-acid sequence alignments and identity/similarity scores were generated using the MUSCLE algorithm in MacVector (version 12.7.5). The three-dimensional (3D) structure of truncated SARS-CoV-2 NCP (comprising the dimerization domain and the C-terminal tail) was predicted using AlphaFold2 (23). Predicted structures were visualized with UCSF Chimera (version 1.13.1) (24) and aligned to the experimentally determined structure of the dimerization domain (25) using the MatchMaker utility.

### Data analysis.

Receiver operating characteristic (ROC) analyses were performed with MedCalc (version 19.2.1); statistical analyses (calculation of 95% confidence intervals [CIs] according to the modified Wald method and Fisher’s exact test) were done using GraphPad Prism QuickCalcs. To avoid statistical bias, a maximum of 1 sample per patient per category was included for calculations of diagnostic and analytical sensitivities. If several samples from an individual patient belonged to the same category, the sample taken on the latest day after the onset of symptoms was included in the analysis.

## RESULTS

### Production of N-terminally truncated SARS-CoV-2 NCP for use as an ELISA antigen.

SARS-CoV-2 NCPΔ246 was recombinantly expressed in E. coli and purified from the soluble fraction of the bacterial lysate (Fig. 1A to C). The amino acid sequence identities of SARS-CoV-2 NCPΔ246 with the respective NCP fragments of other human-pathogenic betacoronaviruses ranged between 25.3% for OC43 and 88.6% for SARS-CoV-1 (Fig. 1D; see also Fig. S1 in the supplemental material).

### Development of FcγR-based SARS-CoV-2 IgG ELISA protocols.

Assay protocols were developed based on the FcγR-based platform technology patented by the Bernhard Nocht Institute for Tropical Medicine (BNITM) (12). Thereby, human sera are coincubated with a biotinylated antigen in a 96-well plate coated with the recombinantly produced immunoglobulin-like extracellular domain of human FcγRIIA (CD32 H131) (14). Subsequently, bound IgG-antigen complexes are detected by the application of HRP-labeled streptavidin and the colorimetric HRP substrate TMB. Two assay versions with different serum and antigen dilutions were validated. In assay version 1 (“ELISA 1”), a final in-well serum dilution of 1:100 (as utilized in the previously developed CCHFV and ZIKV IgG FcγR ELISAs [16, 17]) was employed, while in assay version 2 (“ELISA 2”), a high serum concentration (1:2, final in well) was applied. Biotinylated antigen was titrated at final dilutions in well of 1:20,000, 1:50,000, and 1:75,000 (data not shown); based on the obtained signal-to-noise ratios, final in-well antigen dilutions of 1:20,000 and 1:50,000 for ELISAs 1 and 2, respectively, were chosen.

### Reproducibility.

Intra- and inter-assay variations were assessed using four SARS-CoV-2 IgG-positive serum samples and four *a priori* SARS-CoV-2 IgG-negative serum samples (Table S3). Both ELISAs generated highly reproducible results, with mean intra-assay coefficients of variation (CVs) of <5% and mean inter-assay CVs of <10% for positive signals.

### Detection range and linearity.

**FIG 2:**
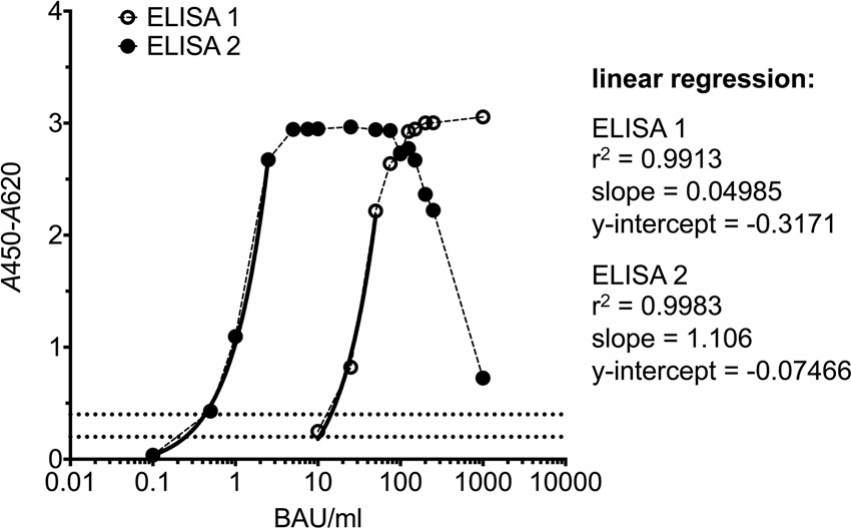
Detection range. WHO international standard plasma 20/136 (1,000 BAU/mL) was serially diluted with serum dilution buffer (SDB) (1× PBS [pH 7.4], 0.05% ProClin 300, 0.01% phenol red) to simulate samples with antibody concentrations ranging between 0.1 BAU/mL and 1,000 BAU/mL. Simulated samples were tested with ELISA 1 (open circles) (final in-well dilutions of 1:100 for samples and 1:20,000 for the conjugate) and ELISA 2 (filled circles) (final in-well dilutions of 1:2 for samples and 1:50,000 for the conjugate). Dotted lines indicate *A*_450_-*A*_620_ values of 0.2 and 0.4, respectively. Solid lines indicate the linear regression fit.

The detection ranges of the two ELISAs were determined using serial dilutions of WHO international standard plasma 20/136 simulating samples with antibody concentrations of between 0.1 BAU/mL and 1,000 BAU/mL (Fig. 2). Assuming an assay cutoff of an *A*_450_-*A*_620_ of 0.2 (see below), ELISA 2 showed a significantly lower detection limit (0.25 U/mL) than ELISA 1 (10.4 U/mL) but exhibited a strong prozone effect at high antibody concentrations (Fig. 2). In particular, signal saturation was observed for ELISA 2 for antibody concentrations of between 7.5 BAU/mL and 75 U/mL; higher antibody concentrations induced a continuous signal drop to an *A*_450_-*A*_620_ of 0.725 for 1,000 BAU/mL.

### Diagnostic sensitivity.

**FIG 3:**
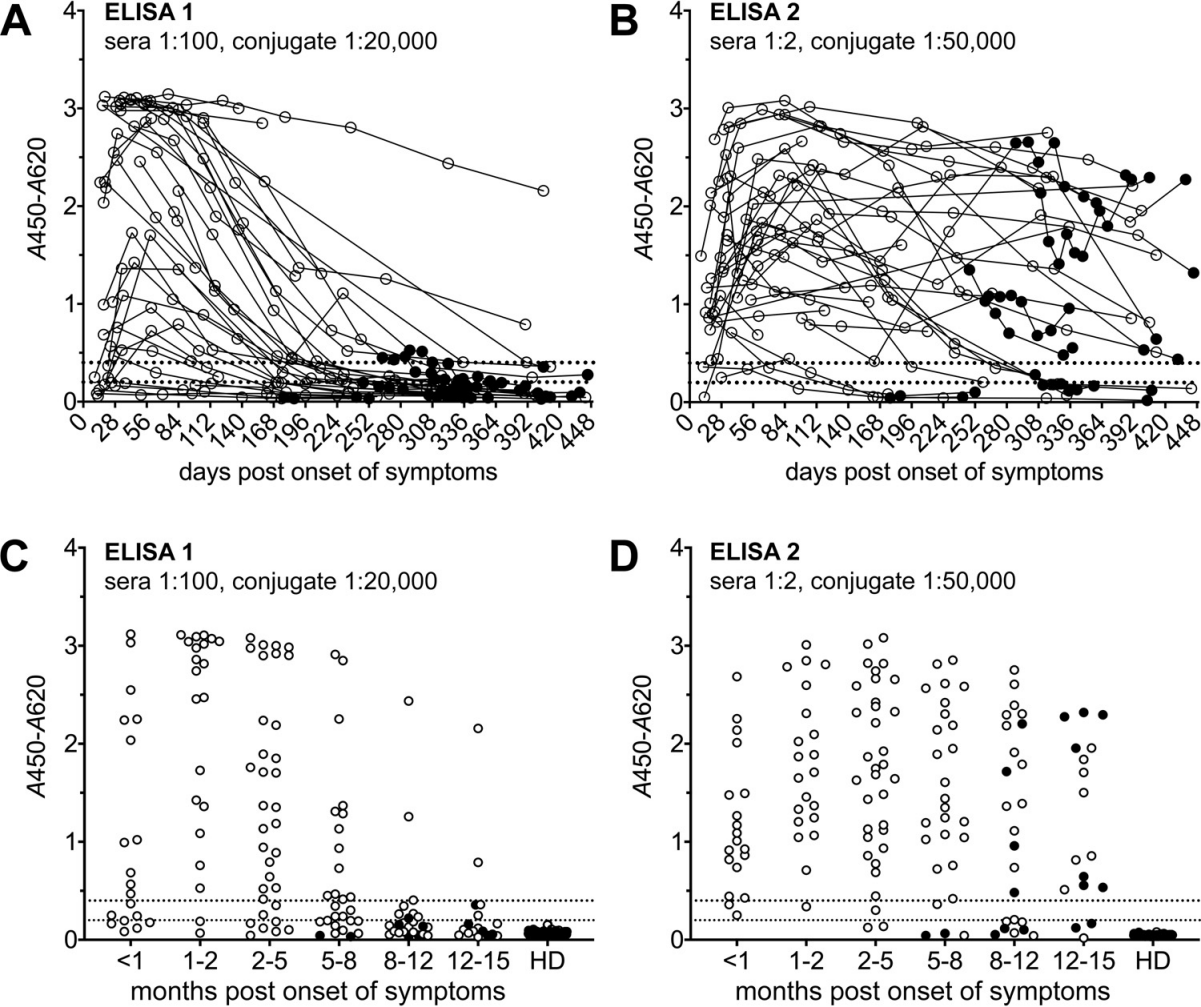
Diagnostic sensitivity. Longitudinal serum samples from 35 COVID-19 patients were analyzed using ELISA 1 (A and C) and ELISA 2 (B and D). (A and B) Trajectories (individual patients; 1 to 13 samples/patient; *n* = 213). (C and D) Stratification according to months after the onset of symptoms (≤1 sample/patient/category; *n* = 146). Dotted lines indicate *A*_450_-*A*_620_ values of 0.2 and 0.4. HD, healthy donors (Germany) (*n* = 139). Filled dots indicate postvaccination samples (spike-based vaccine).

To determine the diagnostic sensitivities of the two SARS-CoV-2 IgG FcγR ELISAs, 213 longitudinal serum samples obtained from 35 German patients with PCR-confirmed SARS-CoV-2 infection and 139 serum samples from healthy German blood donors (HDs), collected before the COVID-19 pandemic, were analyzed (Fig. 3). ROC analyses were performed separately for sample sets collected at different time points after the onset of symptoms (Fig. S2). By applying the lowest possible cutoff value generating 100% specificity in the negative HD panel (*A*_450_-*A*_620_ cutoff = 0.2), diagnostic sensitivities were 90.5% (95% CI, 69.9 to 98.5%) and 100.0% (95% CI, 81.8 to 100.0%) in samples collected 1 to 2 months after the onset of symptoms for ELISAs 1 and 2, respectively (Table 1). While the diagnostic sensitivity of ELISA 1 (sera, 1:100; conjugate, 1:20,000) was found to drop quickly over time in convalescent patients, ELISA 2 (sera, 1:2; conjugate, 1:50,000) enabled the detection of anti-SARS-CoV-2 NCP IgG antibodies for a much longer time period after acute infection (Fig. 3 and Table 1). With the assays being based on NCP antigen, the time course of the humoral response to SARS-CoV-2 infection could still be monitored in individuals having received spike-protein-based vaccines (filled dots in Fig. 3).

**TABLE 1:** Diagnostic sensitivity^*a*^

mpo	dpo	No. of samples	*A*_450_-*A*_620_ cutoff	No. of positive samples, % sensitivity (95% CI) for ELISA 1 (sera, 1:100)	No. of positive samples, % sensitivity (95% CI) for ELISA 2 (sera, 1:2)	*P*
<1	10–28	19	0.200	14, 73.7 (50.9–88.5)	19, 100.0 (80.2–100.0)	0.0463
			0.400	11, 57.9 (36.2–76.9)	17, 89.5 (67.4–98.3)	0.0625

1–2	29–56	21	0.200	19, 90.5 (69.9–98.5)	21, 100.0 (81.8–100.0)	0.4878
			0.400	19, 90.5 (69.9–98.5)	20, 95.2 (75.6–100.0)	1.0000

2–5	57–140	34	0.200	28, 82.3 (66.1–92.0)	32, 94.1 (79.9–99.3)	0.2585
			0.400	26, 76.5 (59.8–87.8)	31, 91.2 (76.3–97.7)	0.1863

5–8	141–224	27	0.200	17, 63.0 (44.2–78.5)	24, 88.9 (71.1–96.8)	0.0537
			0.400	13, 48.1 (30.7–66.0)	23, 85.2 (66.9–94.7)	0.0084

8–12	225–336	24	0.200	8, 33.3 (17.8–53.4)	17, 70.8 (50.6–85.3)	0.0199
			0.400	3, 12.5 (3.5–31.8)	16, 66.7 (46.6–82.2)	0.0003

12–15	337–396	17	0.200	5, 29.4 (13.0–53.4)	14, 82.3 (58.2–94.6)	0.0049
			0.400	2, 11.8 (2.0–35.6)	14, 82.3 (58.2–94.6)	0.0001

a Longitudinal serum samples obtained from 35 COVID-19 patients were stratified according to months postonset (mpo) (≤1 sample/patient/category). Sensitivities as well as 95% confidence intervals (CIs) were calculated for two alternative cutoff values (*A*_450_-*A*_620_ = 0.2 and 0.4). dpo, days postonset. *P* values were determined using Fisher’s exact test.

### Analytical sensitivity.

**FIG 4:**
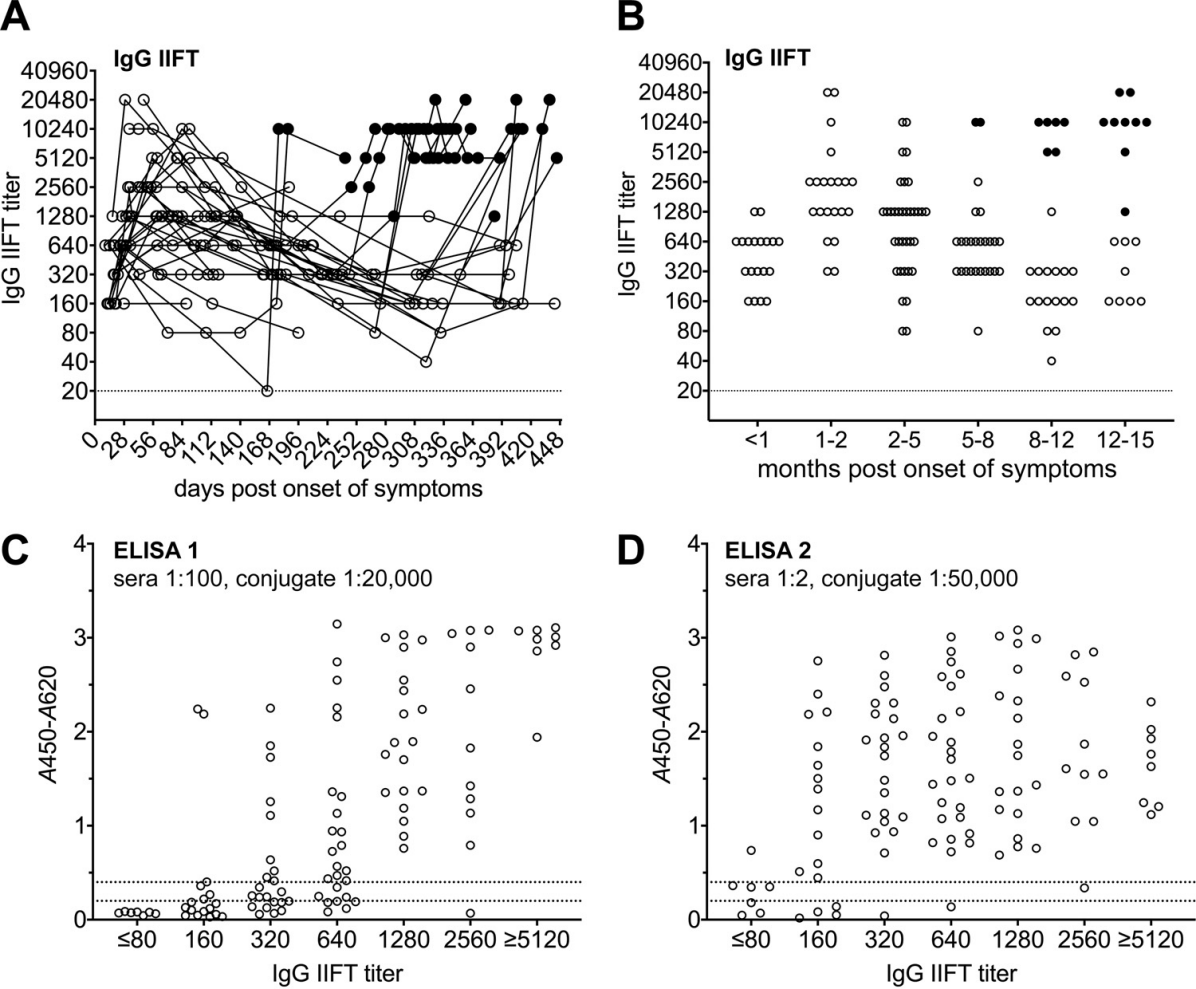
Analytical sensitivity. (A and B) Longitudinal serum samples from 35 COVID-19 patients were analyzed by IgG IIFT. Filled dots indicate postvaccination samples (spike-based vaccine). (A) Trajectories (individual patients; 1 to 13 samples/patient; *n* = 213); (B) stratification according to months after the onset of symptoms (≤1 sample/patient/category; *n* = 146). (C and D) Results of ELISA 1 (C) and ELISA 2 (D) for prevaccination samples stratified according to IIFT titers (≤1 sample/patient/category; *n* = 109). Dotted lines indicate *A*_450_-*A*_620_ values of 0.2 and 0.4.

Immunofluorescence analysis of COVID-19 patient samples using SARS-CoV-2-infected Vero cells revealed the highest IgG antibody titers between 1 and 2 months after the onset of symptoms (Fig. 4A and B). Later in the convalescent phase, the median IgG antibody titers dropped considerably but persisted at detectable levels in most patients for at least 12 months; spike-based vaccines strongly boosted SARS-CoV-2 IgG IIFT titers (filled dots in Fig. 4A and B). To determine the analytical sensitivities of the two SARS-CoV-2 IgG FcγR ELISAs in relation to IIFT (serving as a highly sensitive gold-standard antibody binding assay), 109 samples from 35 patients with PCR-confirmed SARS-CoV-2 infection taken prior to an eventual vaccination with a spike-based vaccine were stratified according to their respective IgG IIFT titers (≤1 sample/patient/category; median/range, 123/13 to 443 dpo). While samples with a high IIFT titer of SARS-CoV-2 IgG antibodies (≥1,280) were readily detected as positive by both ELISAs, the analytical sensitivity was significantly higher for ELISA 2 when analyzing samples with an IgG IIFT titer of between 160 and 640 (Fig. 4C and D and Table 2). In samples with a high SARS-CoV-2 IgG IIFT titer (≥5,120), a prozone effect was observed for SARS-CoV-2 IgG FcγR ELISA 2 (Fig. 4D).

**TABLE 2:** Analytical sensitivity^*a*^

IgG IIFT titer	No. of samples	*A*_450_-*A*_620_ cutoff	No. of positive samples, % sensitivity (95% CI), for ELISA 1 (sera, 1:100)	No. of positive samples, % sensitivity (95% CI), for ELISA 2 (sera, 1:2)	*P*
≤80	7	0.200	0, 0.0 (0.0–40.4)	4, 57.1 (25.0–84.2)	0.0699
		0.400	0, 0.0 (0.0–40.4)	1, 14.3 (0.5–53.3)	1.0000

160	17	0.200	6, 35.3 (17.2–58.8)	13, 76.5 (52.2–90.9)	0.0366
		0.400	3, 17.6 (5.4–41.8)	13, 76.5 (52.2–90.9)	0.0016

320	22	0.200	14, 63.6 (42.9–80.4)	21, 95.4 (76.5–100.0)	0.0212
		0.400	9, 40.9 (23.2–61.3)	21, 95.4 (76.5–100.0)	0.0002

640	25	0.200	20, 80.0 (60.4–91.6)	24, 96.0 (78.9–100.0)	0.1895
		0.400	17, 68.0 (48.3–82.9)	24, 96.0 (78.9–100.0)	0.0232

1,280	19	0.200	19, 100.0 (80.2–100.0)	19, 100.0 (80.2–100.0)	1.0000
		0.400	19, 100.0 (80.2–100.0)	19, 100.0 (80.2–100.0)	1.0000

2,560	11	0.200	10, 90.9 (60.1–100.0)	11, 100.0 (70.0–100.0)	1.0000
		0.400	10, 90.9 (60.1–100.0)	10, 90.9 (60.1–100.0)	1.0000

≥5,120	8	0.200	8, 100.0 (62.8–100.0)	8, 100.0 (62.8–100.0)	1.0000
		0.400	8, 100.0 (62.8–100.0)	8, 100.0 (62.8–100.0)	1.0000

a Longitudinal serum samples obtained from 35 COVID-19 patients were stratified according to SARS-CoV-2 IgG IIFT titers (≤1 sample/patient/category). Sensitivities as well as 95% confidence intervals (CIs) were calculated for two alternative cutoff values (*A*_450_-*A*_620_ = 0.2 and 0.4). *P* values were determined using Fisher’s exact test.

Comparative testing of a subset of 82 IIFT positive samples from 26 COVID-19 patients (dpo range: 10 to 178) with a commercially available, NCP-based indirect ELISA (Euroimmun SARS-CoV-2 NCP IgG) revealed a high concordance with SARS-CoV-2 FcγR IgG ELISA 1 (Fig. S3).

### Specificity.

The specificities of the two SARS-CoV-2 IgG FcγR ELISAs in comparison with the Euroimmun SARS-CoV-2 IgG NCP ELISA (Table 3; Fig. S4) were evaluated using *a priori* SARS-CoV-2 IgG-negative serum panels acquired from symptom-free donors before the COVID-19 pandemic in Europe (Germany), Africa (Ghana, Madagascar, and Nigeria), Asia (Lao PDR), and South America (Colombia) (Table S2) (3). While false-positive rates were low for both the commercially available ELISA and the newly developed tests with the European serum panel, both SARS-CoV-2 IgG FcγR ELISAs showed superior specificity in application to African serum panels (Table 3; Fig. S4).

**TABLE 3:** Specificity of SARS-CoV-2 IgG ELISAs^*a*^

Panel	No. of samples	No. (%) of betacoronavirus IgG-positive samples	*A*_450_-*A*_620_ cutoff	No. of negative samples, % specificity (95% CI), for ELISA 1 (sera, 1:100)	No. of negative samples, % specificity (95% CI), for ELISA 2 (sera, 1:2)	No. of negative/bl samples, % specificity (95% CI), for Euroimmun NCP IgG
Germany	139	51 (36.7)	0.200	139, 100.0 (96.8–100.0)	139, 100.0 (96.8–100.0)	139, 100.0 (96.8–100.0)
			0.400	139, 100.0 (96.8–100.0)	139, 100.0 (96.8–100.0)	

Ghana A	131	33 (25.2)	0.200	128, 97.7 (93.2–99.5)	129, 98.5 (94.3–99.9)	97, 74.0 (65.9–80.8)
			0.400	129, 98.5 (94.3–99.9)	131, 100.0 (96.6–100.0)	

Ghana B	145	34 (23.4)	0.200	142, 97.9 (93.8–99.6)	145, 100.0 (96.9–100.0)	132, 91.0 (85.1–94.8)
			0.400	145, 100.0 (96.9–100.0)	145, 100.0 (96.9–100.0)	

Madagascar	166	30 (18.1)	0.200	165, 99.4 (96.3–100.0)	166, 100.0 (97.3–100.0)	164, 98.8 (95.4–100.0)
			0.400	166, 100.0 (97.3–100.0)	166, 100.0 (97.3–100.0)	

Nigeria	149	79 (53.0)	0.200	143, 96.0 (91.3–98.3)	149, 100.0 (97.0–100.0)	107, 71.8 (64.1–78.4)
			0.400	146, 98.0 (94.0–99.6)	149, 100.0 (97.0–100.0)	

Colombia	40	12 (30.0)	0.200	40, 100.0 (89.6–100.0)	40, 100.0 (89.6–100.0)	40, 100.0 (89.6–100.0)
			0.400	40, 100.0 (89.6–100.0)	40, 100.0 (89.6–100.0)	

Lao PDR	20	5 (25.0)	0.200	20, 100.0 (81.0–100.0)	20, 100.0 (81.0–100.0)	20, 100.0 (81.0–100.0)
			0.400	20, 100.0 (81.0–100.0)	20, 100.0 (81.0–100.0)	

a Serum/plasma samples collected from symptom-free donors before 2019 in Europe, Africa, South America, and Asia were analyzed with SARS-CoV-2 IgG FcγR ELISAs 1 and 2 and the Euroimmun anti-SARS-CoV-2 NCP ELISA (IgG). Specificities and 95% confidence intervals (CIs) were calculated for two alternative cutoff values (*A*_450_-*A*_620_ = 0.2 and 0.4). bl, borderline.

Although IgG antibodies interacting with the corresponding NCP fragments of the betacoronaviruses OC43 and/or HKU1 (Table S1) were detected in 244 (31%) of 790 tested serum samples (Table 3; Fig. S5) (3), no obvious cross-reactivity of these antibodies with the SARS-CoV-2 antigen has been observed. Indeed, false-positive signals obtained for a small number of Nigerian serum samples with SARS-CoV-2 IgG FcγR ELISA 1 could not be suppressed with an excess of unlabeled OC43 NCP, although high levels of antibodies interacting with this protein were present in these samples (data not shown).

### Interference.

For SARS-CoV-2 IgG FcγR ELISA 1 (sera, 1:100, final in well; antigen, 1:20,000, final in well), no significant influence on the obtained *A*_450_-*A*_620_ values was observed for final serum concentrations of 10 mg/mL hemoglobin, 0.5 mg/mL bilirubin, and 5 mg/mL triglycerides and biotin concentrations of up to 100,000 ng/mL. While a rheumatoid factor serum concentration of 50 IU/mL did not influence the signal height, concentrations of 100 IU/mL and 1,000 IU/mL suppressed positive signals by ca. 30% and 50%, respectively, without increasing the assay background.

For SARS-CoV-2 IgG FcγR ELISA 2 (sera, 1:2, final in well; antigen, 1:50,000, final in well), although a final biotin serum concentration of 100,000 ng/mL induced a significant signal loss for positive sera, a concentration of 3,500 ng/mL did not significantly influence *A*_450_-*A*_620_ values. Hemoglobin serum concentrations of up to 5 mg/mL did not influence assay performance, but signals were impacted at 10 mg/mL. Rheumatoid factor serum concentrations of 50 IU/mL and 1,000 IU/mL reduced the intensities of positive signals by ca. 40%, and 50%, respectively, without increasing the assay background. The influence of bilirubin and triglycerides could not be studied for ELISA 2 due to the strong impact of the solvent used (chloroform) on antigen integrity.

## DISCUSSION

### Assay protocol.

The SARS-CoV-2 IgG ELISAs developed and validated in this work employ a patented platform technology (12, 14–17) in which immune complexes between a labeled, recombinant antigen and human serum IgG antibodies are formed in solution and subsequently captured by an isotype-specific FcγR immunoglobulin-like domain. Thereby, the use of a soluble, natively folded antigen has a direct positive impact on sensitivity and specificity because conformational epitopes are preserved and nonspecific interactions with hydrophobic amino acid stretches that may be exposed in incorrectly folded proteins cannot occur. With the main intended use of our assays being the monitoring of seroconversion and seroprevalence in archived samples, development was focused on the optimization of sensitivity and specificity rather than a minimized time to result. Nevertheless, preliminary results indicate that upon adjustments of conjugate and serum concentrations, shortening the antigen-sample incubation step from overnight to 2 h might be possible, with only minor losses in sensitivity and no increase in the assay background (data not shown). Importantly, the assays, which are procurable via the European Virus Archive Goes Global (EVAg) webpage (26), can be performed manually, and results are generated using a standard absorbance ELISA reader, facilitating their use also in settings where no highly specialized technical equipment is available.

### Choice of antigen.

As an antigen, a SARS-CoV-2 NCP fragment comprising the dimerization and C-terminal tail domains of SARS-CoV-2 NCP (see Fig. S1 in the supplemental material) was used. Similar fragments of SARS-CoV-1 NCP have previously been shown to allow the sensitive detection of anti-SARS-CoV-1 IgG antibodies in patient sera (27, 28) while generating fewer false-positive responses than the full-length protein in negative-control sera (28). As for other viral nucleocapsid proteins (16, 28), the cost-efficient production of large quantities of natively folded SARS-CoV-2 full-length NCP and subdomains is possible in E. coli (7).

### Assay sensitivity.

Complete validation was performed for two SARS-CoV-2 IgG FcγR ELISA protocols employing different final serum and conjugate dilutions (ELISA 1, sera at 1:100 and conjugate at 1:20,000; ELISA 2, sera at 1:2 and conjugate at 1:50,000). ELISA 1 displayed diagnostic and analytical sensitivities similar to those of a commercially available, manually performed, plate-based indirect ELISA (Euroimmun SARS-CoV-2 NCP IgG ELISA) (29), thus showing a significant waning of signal intensity in COVID-19 patients at approximately 6 months postinfection. In contrast, ELISA 2 allowed the persistent detection of anti-NCP IgG antibodies for more than 12 months postinfection for most patients. Thus, SARS-CoV-2 IgG FcγR ELISA 2 displays a sensitivity comparable to that of Elecsys anti-SARS-CoV-2 (Roche), a commercially available NCP-based automated electrochemiluminescence immunoassay system detecting total Ig through double-antigen binding (30, 31).

### Assay specificity.

As we have shown recently, unacceptably high false-positive rates of up to 61% are obtained with several commercially available indirect SARS-CoV-2 NCP IgG ELISAs in African sample panels collected before the COVID-19 pandemic, although the very same assays displayed excellent specificity in European samples (3). Therefore, the specificity of the newly developed SARS-CoV-2 IgG FcγR ELISAs was challenged with an extensive panel of 790 pre-COVID-19 negative-control samples originating from Europe (*n* = 139), South America (*n* = 40), Asia (*n* = 20), and Africa (*n* = 591). Both ELISAs were found to be highly specific (>96%), even in the most challenging sample panel, originating from Nigeria. For applications requiring maximum specificity in African samples, we recommend using a slightly elevated cutoff value of an *A*_450_-*A*_620_ of 0.4 (instead of 0.2 for samples from European residents). As for the commercially available assays evaluated in our previous study (3), the presence of IgG antibodies elicited by previous infections with the common cold CoVs OC43, HKU1, NL63, and 229E did not *per se* compromise SARS-CoV-2 IgG FcγR ELISA specificity. Indeed, in concordance with data from previous studies (1, 32), those antibodies were found in a significant proportion of donor sera of each origin (Table 3; Fig. S5). Three Nigerian samples generated false-positive signals in ELISA 1 but not ELISA 2. Although high levels of anti-OC43 IgG antibodies were detected in all three serum samples, signals in the SARS-CoV-2 IgG FcγR ELISA could not be suppressed by the addition of excess OC43 NCP, ruling out OC43 cross-reactivity as a cause. A possible explanation for these false-positive signals occurring in ELISA 1 but not ELISA 2 could be the presence of high levels of IgG antibodies elicited by another, as-yet-unidentified member of the *Coronaviridae* family binding with low affinity to SARS-CoV-2 NCP.

### Caveats and limitations.

When applying the SARS-CoV-2 IgG FcγR ELISAs presented in this study in seroepidemiological research projects, several caveats and limitations have to be considered. First of all, due to the use of SARS-CoV-2 NCP as the antigen, humoral immune responses elicited by spike-based vaccines (e.g., BioNTech/Pfizer and AstraZeneca) are not detected by these assays. Correspondingly, detection of natural SARS-CoV-2 infection is possible in naive individuals and probands vaccinated with spike-based vaccines but not with vaccines based on inactivated full virus (e.g., Sinopharm and Sinovac). The assay version should be chosen according to the respective research questions: while ELISA 1 is most suitable to monitor the development of the humoral immune response during the acute and early convalescent phases of the disease, ELISA 2 allows the detection of SARS-CoV-2-specific antibodies for a much longer time period postinfection and therefore is the assay version of choice for large-scale seroprevalence studies (30). Nevertheless, this assay shows a strong prozone effect in serum samples with very high anti-NCP IgG titers. Therefore, misleadingly low readings could occur in certain situations, such as during early convalescent stages of severe infections (1) or upon reinfections/vaccinations strongly boosting the anti-NCP response. If these scenarios are likely, the parallel performance of ELISAs 1 and 2 is strongly recommended. Furthermore, ELISA 2 is (due to the direct use of pure, undiluted serum samples) more susceptible to interfering substances than ELISA 1. Therefore, serum quality should be monitored and critically assessed, particularly when using this assay version. Further potential limitations, relevant for both assay versions, are the differential affinities of the recombinant capture molecule CD32 H131 for the various IgG subclasses (33) and the potential masking of relevant epitopes by the biotin label. Nevertheless, the dominant IgG subclasses induced by SARS-CoV-2 infection, IgG1 and IgG3 (34, 35), have been shown to strongly interact with CD32 H131 in a previous study (33).

## References

[1] Hamady A, Lee J, Loboda ZA. 2022. Waning antibody responses in COVID-19: what can we learn from the analysis of other coronaviruses? Infection 50:11–25. doi:10.1007/s15010-021-01664-z. 34324165 PMC8319587

[2] Deeks JJ, Dinnes J, Takwoingi Y, Davenport C, Spijker R, Taylor-Phillips S, Adriano A, Beese S, Dretzke J, Ferrante di Ruffano L, Harris IM, Price MJ, Dittrich S, Emperador D, Hooft L, Leeflang MMG, Van den Bruel A, Cochrane COVID-19 Diagnostic Test Accuracy Group. 2020. Antibody tests for identification of current and past infection with SARS-CoV-2. Cochrane Database Syst Rev 6:CD013652. doi:10.1002/14651858.CD013652. 32584464 PMC7387103

[3] Emmerich P, Murawski C, Ehmen C, von Possel R, Pekarek N, Oestereich L, Duraffour S, Pahlmann M, Struck N, Eibach D, Krumkamp R, Amuasi J, Maiga‐Ascofaré O, Rakotozandrindrainy R, Asogun D, Ighodalo Y, Kann S, May J, Tannich E, Deschermeier C. 2021. Limited specificity of commercially available SARS-CoV-2 IgG ELISAs in serum samples of African origin. Trop Med Int Health 26:621–631. doi:10.1111/tmi.13569. 33666297 PMC8014856

[4] Nkuba Ndaye A, Hoxha A, Madinga J, Marien J, Peeters M, Leendertz FH, Ahuka Mundeke S, Arien KK, Muyembe Tanfumu J-J, Mbala Kingebeni P, Vanlerberghe V. 2021. Challenges in interpreting SARS-CoV-2 serological results in African countries. Lancet Glob Health 9:e588–e589. doi:10.1016/S2214-109X(21)00060-7. 33609481 PMC7906714

[5] Yadouleton A, Sander A-L, Moreira-Soto A, Tchibozo C, Hounkanrin G, Badou Y, Fischer C, Krause N, Akogbeto P, de Oliveira Filho EF, Dossou A, Brunink S, Aissi MAJ, Djingarey MH, Hounkpatin B, Nagel M, Drexler JF. 2021. Limited specificity of serologic tests for SARS-CoV-2 antibody detection, Benin. Emerg Infect Dis 27:233–237. doi:10.3201/eid2701.203281. PMC7774555 33261717

[6] Lustig Y, Keler S, Kolodny R, Ben-Tal N, Atias-Varon D, Shlush E, Gerlic M, Munitz A, Doolman R, Asraf K, Shlush LI, Vivante A. 2021. Potential antigenic cross-reactivity between severe acute respiratory syndrome coronavirus 2 (SARS-CoV-2) and dengue viruses. Clin Infect Dis 73:e2444–e2449. doi:10.1093/cid/ciaa1207. 32797228 PMC7454334

[7] Ye Q, West AMV, Silletti S, Corbett KD. 2020. Architecture and self-assembly of the SARS-CoV-2 nucleocapsid protein. Protein Sci 29:1890–1901. doi:10.1002/pro.3909. 32654247 PMC7405475

[8] Allen N, Brady M, Carrion Martin AI, Domegan L, Walsh C, Doherty L, Riain UN, Bergin C, Fleming C, Conlon N. 2021. Serological markers of SARS-CoV-2 infection; anti-nucleocapsid antibody positivity may not be the ideal marker of natural infection in vaccinated individuals. J Infect 83:e9–e10. doi:10.1016/j.jinf.2021.08.012. PMC8351117 34384812

[9] Hachim A, Kavian N, Cohen CA, Chin AWH, Chu DKW, Mok CKP, Tsang OTY, Yeung YC, Perera R, Poon LLM, Peiris JSM, Valkenburg SA. 2020. ORF8 and ORF3b antibodies are accurate serological markers of early and late SARS-CoV-2 infection. Nat Immunol 21:1293–1301. doi:10.1038/s41590-020-0773-7. 32807944

[10] Lam JY, Yuen CK, Ip JD, Wong WM, To KK, Yuen KY, Kok KH. 2020. Loss of orf3b in the circulating SARS-CoV-2 strains. Emerg Microbes Infect 9:2685–2696. doi:10.1080/22221751.2020.1852892. 33205709 PMC7782295

[11] Pereira F. 2021. SARS-CoV-2 variants lacking a functional ORF8 may reduce accuracy of serological testing. J Immunol Methods 488:112906. doi:10.1016/j.jim.2020.112906. 33137303 PMC7604215

[12] Bernhard Nocht Institute for Tropical Medicine. 2013. European patent EP2492689.

[13] Bernhard Nocht Institute for Tropical Medicine. 2020. European patent EP3207375.

[14] Emmerich P, Mika A, Schmitz H. 2013. Detection of serotype-specific antibodies to the four dengue viruses using an immune complex binding (ICB) ELISA. PLoS Negl Trop Dis 7:e2580. doi:10.1371/journal.pntd.0002580. 24386498 PMC3873247

[15] Gabriel M, Adomeh DI, Ehimuan J, Oyakhilome J, Omomoh EO, Ighodalo Y, Olokor T, Bonney K, Pahlmann M, Emmerich P, Lelke M, Brunotte L, Olschlager S, Thome-Bolduan C, Becker-Ziaja B, Busch C, Odia I, Ogbaini-Emovon E, Okokhere PO, Okogbenin SA, Akpede GO, Schmitz H, Asogun DA, Gunther S. 2018. Development and evaluation of antibody-capture immunoassays for detection of Lassa virus nucleoprotein-specific immunoglobulin M and G. PLoS Negl Trop Dis 12:e0006361. doi:10.1371/journal.pntd.0006361. 29596412 PMC5892945

[16] Emmerich P, Mika A, von Possel R, Rackow A, Liu Y, Schmitz H, Gunther S, Sherifi K, Halili B, Jakupi X, Berisha L, Ahmeti S, Deschermeier C. 2018. Sensitive and specific detection of Crimean-Congo hemorrhagic fever virus (CCHFV)-specific IgM and IgG antibodies in human sera using recombinant CCHFV nucleoprotein as antigen in mu-capture and IgG immune complex (IC) ELISA tests. PLoS Negl Trop Dis 12:e0006366. doi:10.1371/journal.pntd.0006366. 29579040 PMC5892944

[17] Ehmen C, Medialdea-Carrera R, Brown D, de Filippis AMB, de Sequeira PC, Nogueira RMR, Brasil P, Calvet GA, Blessmann J, Mallmann AM, Sievertsen J, Rackow A, Schmidt-Chanasit J, Emmerich P, Schmitz H, Deschermeier C, Mika A. 2021. Accurate detection of Zika virus IgG using a novel immune complex binding ELISA. Trop Med Int Health 26:89–101. doi:10.1111/tmi.13505. 33012038

[18] Rackow A, Ehmen C, von Possel R, Medialdea-Carrera R, Brown D, Bispo de Filippis AM, Carvalho de Sequeira P, Ribeiro Nogueira RM, Halili B, Jakupi X, Berisha L, Ahmeti S, Sherifi K, Schmidt-Chanasit J, Schmitz H, Mika A, Emmerich P, Deschermeier C. 2019. Immunoglobulin-like domain of HsFcmuR as a capture molecule for detection of Crimean-Congo hemorrhagic fever virus- and Zika virus-specific IgM antibodies. Clin Chem 65:451–461. doi:10.1373/clinchem.2018.294819. 30709812

[19] Schmitz H, Gabriel M, Emmerich P. 2011. Specific detection of antibodies to different flaviviruses using a new immune complex ELISA. Med Microbiol Immunol 200:233–239. doi:10.1007/s00430-011-0195-0. 21533786

[20] Berrow NS, Alderton D, Sainsbury S, Nettleship J, Assenberg R, Rahman N, Stuart DI, Owens RJ. 2007. A versatile ligation-independent cloning method suitable for high-throughput expression screening applications. Nucleic Acids Res 35:e45. doi:10.1093/nar/gkm047. 17317681 PMC1874605

[21] WHO Working Group on the Clinical Characterisation and Management of COVID-19 Infection. 2020. A minimal common outcome measure set for COVID-19 clinical research. Lancet Infect Dis 20:e192–e197. doi:10.1016/S1473-3099(20)30483-7. 32539990 PMC7292605

[22] Reisinger EC, von Possel R, Warnke P, Geerdes-Fenge HF, Hemmer CJ, Pfefferle S, Lobermann M, Littmann M, Emmerich P. 2020. Screening of mothers in a COVID-19 low-prevalence region: determination of SARS-CoV-2 antibodies in 401 mothers from Rostock by ELISA and confirmation by immunofluorescence. Dtsch Med Wochenschr 145:e96–e100. (In German). doi:10.1055/a-1197-4293. 32572869 PMC7446142

[23] Jumper J, Evans R, Pritzel A, Green T, Figurnov M, Ronneberger O, Tunyasuvunakool K, Bates R, Zidek A, Potapenko A, Bridgland A, Meyer C, Kohl SAA, Ballard AJ, Cowie A, Romera-Paredes B, Nikolov S, Jain R, Adler J, Back T, Petersen S, Reiman D, Clancy E, Zielinski M, Steinegger M, Pacholska M, Berghammer T, Bodenstein S, Silver D, Vinyals O, Senior AW, Kavukcuoglu K, Kohli P, Hassabis D. 2021. Highly accurate protein structure prediction with AlphaFold. Nature 596:583–589. doi:10.1038/s41586-021-03819-2. 34265844 PMC8371605

[24] Pettersen EF, Goddard TD, Huang CC, Couch GS, Greenblatt DM, Meng EC, Ferrin TE. 2004. UCSF Chimera—a visualization system for exploratory research and analysis. J Comput Chem 25:1605–1612. doi:10.1002/jcc.20084. 15264254

[25] Zhou R, Zeng R, von Brunn A, Lei J. 2020. Structural characterization of the C-terminal domain of SARS-CoV-2 nucleocapsid protein. Mol Biomed 1:2. doi:10.1186/s43556-020-00001-4. PMC7406681 34765991

[26] Romette JL, Prat CM, Gould EA, de Lamballerie X, Charrel R, Coutard B, Fooks AR, Bardsley M, Carroll M, Drosten C, Drexler JF, Gunther S, Klempa B, Pinschewer D, Klimkait T, Avsic-Zupanc T, Capobianchi MR, Dicaro A, Ippolito G, Nitsche A, Koopmans M, Reusken C, Gorbalenya A, Raoul H, Bourhy H, Mettenleiter T, Reiche S, Batten C, Sabeta C, Paweska JT, Eropkin M, Zverev V, Hu Z, Mac Cullough S, Mirazimi A, Pradel F, Lieutaud P. 2018. The European Virus Archive Goes Global: a growing resource for research. Antiviral Res 158:127–134. doi:10.1016/j.antiviral.2018.07.017. 30059721 PMC7127435

[27] Chen S, Lu D, Zhang M, Che J, Yin Z, Zhang S, Zhang W, Bo X, Ding Y, Wang S. 2005. Double-antigen sandwich ELISA for detection of antibodies to SARS-associated coronavirus in human serum. Eur J Clin Microbiol Infect Dis 24:549–553. doi:10.1007/s10096-005-1378-7. 16133409 PMC7088218

[28] Yu F, Le MQ, Inoue S, Thai HTC, Hasebe F, Del Carmen Parquet M, Morita K. 2005. Evaluation of inapparent nosocomial severe acute respiratory syndrome coronavirus infection in Vietnam by use of highly specific recombinant truncated nucleocapsid protein-based enzyme-linked immunosorbent assay. Clin Diagn Lab Immunol 12:848–854. doi:10.1128/CDLI.12.7.848-854.2005. 16002634 PMC1182204

[29] Emmerich P, von Possel R, Hemmer CJ, Fritzsche C, Geerdes-Fenge H, Menge B, Messing C, Borchardt-Loholter V, Deschermeier C, Steinhagen K. 2021. Longitudinal detection of SARS-CoV-2-specific antibody responses with different serological methods. J Med Virol 93:5816–5824. doi:10.1002/jmv.27113. 34061367 PMC8242665

[30] Di Germanio C, Simmons G, Kelly K, Martinelli R, Darst O, Azimpouran M, Stone M, Hazegh K, Grebe E, Zhang S, Ma P, Orzechowski M, Gomez JE, Livny J, Hung DT, Vassallo R, Busch MP, Dumont LJ. 2021. SARS-CoV-2 antibody persistence in COVID-19 convalescent plasma donors: dependency on assay format and applicability to serosurveillance. Transfusion 61:2677–2687. doi:10.1111/trf.16555. 34121205 PMC8447038

[31] Theel ES, Johnson PW, Kunze KL, Wu L, Gorsh AP, Granger D, Roforth MM, Jerde CR, Lasho M, Andersen KJ, Murphy BM, Harring J, Lake DF, Svarovsky SA, Senefeld JW, Carter RE, Joyner MJ, Baumann NA, Mills JR. 2021. SARS-CoV-2 serologic assays dependent on dual-antigen binding demonstrate diverging kinetics relative to other antibody detection methods. J Clin Microbiol 59:e01231-21. doi:10.1128/JCM.01231-21. PMC8373029 34166066

[32] Zhou W, Wang W, Wang H, Lu R, Tan W. 2013. First infection by all four non-severe acute respiratory syndrome human coronaviruses takes place during childhood. BMC Infect Dis 13:433. doi:10.1186/1471-2334-13-433. 24040960 PMC3848659

[33] Bruhns P, Iannascoli B, England P, Mancardi DA, Fernandez N, Jorieux S, Daeron M. 2009. Specificity and affinity of human Fcgamma receptors and their polymorphic variants for human IgG subclasses. Blood 113:3716–3725. doi:10.1182/blood-2008-09-179754. 19018092

[34] Luo H, Jia T, Chen J, Zeng S, Qiu Z, Wu S, Li X, Lei Y, Wang X, Wu W, Zhang R, Zou X, Feng T, Ding R, Zhang Y, Chen YQ, Sun C, Wang T, Fang S, Shu Y. 2021. The characterization of disease severity associated IgG subclasses response in COVID-19 patients. Front Immunol 12:632814. doi:10.3389/fimmu.2021.632814. 33763078 PMC7982848

[35] Yates JL, Ehrbar DJ, Hunt DT, Girardin RC, Dupuis AP, II, Payne AF, Sowizral M, Varney S, Kulas KE, Demarest VL, Howard KM, Carson K, Hales M, Ejemel M, Li Q, Wang Y, Peredo-Wende R, Ramani A, Singh G, Strle K, Mantis NJ, McDonough KA, Lee WT. 2021. Serological analysis reveals an imbalanced IgG subclass composition associated with COVID-19 disease severity. Cell Rep Med 2:100329. doi:10.1016/j.xcrm.2021.100329. 34151306 PMC8205277

