# Supplementary material for: Fcγ-Receptor-Based Enzyme-Linked Immunosorbent Assays for Sensitive, Specific, and Persistent Detection of Anti-SARS-CoV-2 Nucleocapsid Protein IgG Antibodies in Human Sera
Source: J Clin Microbiol. 2022 May 16;60(6):e00075-22. doi: 10.1128/jcm.00075-22 (PMC9199419; doi:10.1128/jcm.00075-22)
Supplement: Supplemental file 1 — Fig. S1 to S5. Download jcm.00075-22-s0001.pdf, PDF file, 7.5 MB [file jcm.00075-22-s0001.pdf]

Supplemental Figure 1

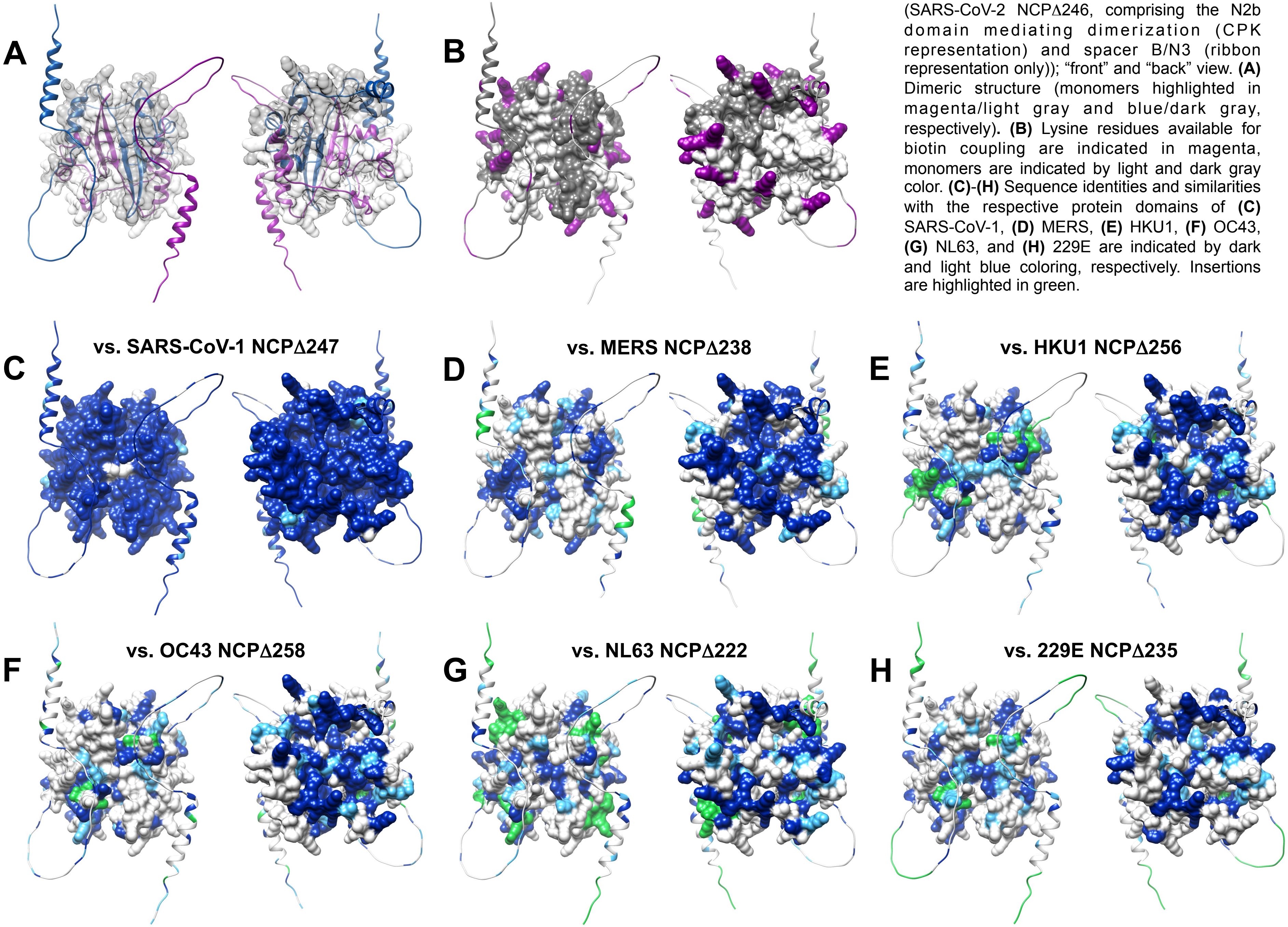

Supplemental Fig. 1. Structure model of N-terminally truncated SARS-CoV-2 NCP (SARS-CoV-2 NCPΔ246, comprising the N2b domain mediating dimerization (CPK representation) and spacer B/N3 (ribbon representation only)); "front" and "back" view. **(A)** Dimeric structure (monomers highlighted in magenta/light gray and blue/dark gray, respectively)**.** **(B)** Lysine residues available for biotin coupling are indicated in magenta, monomers are indicated by light and dark gray color. **(C)-(H)** Sequence identities and similarities with the respective protein domains of **(C)** SARS-CoV-1, **(D)** MERS, **(E)** HKU1, **(F)** OC43, **(G)** NL63, and **(H)** 229E are indicated by dark and light blue coloring, respectively. Insertions are highlighted in green.

Supplemental Figure 2

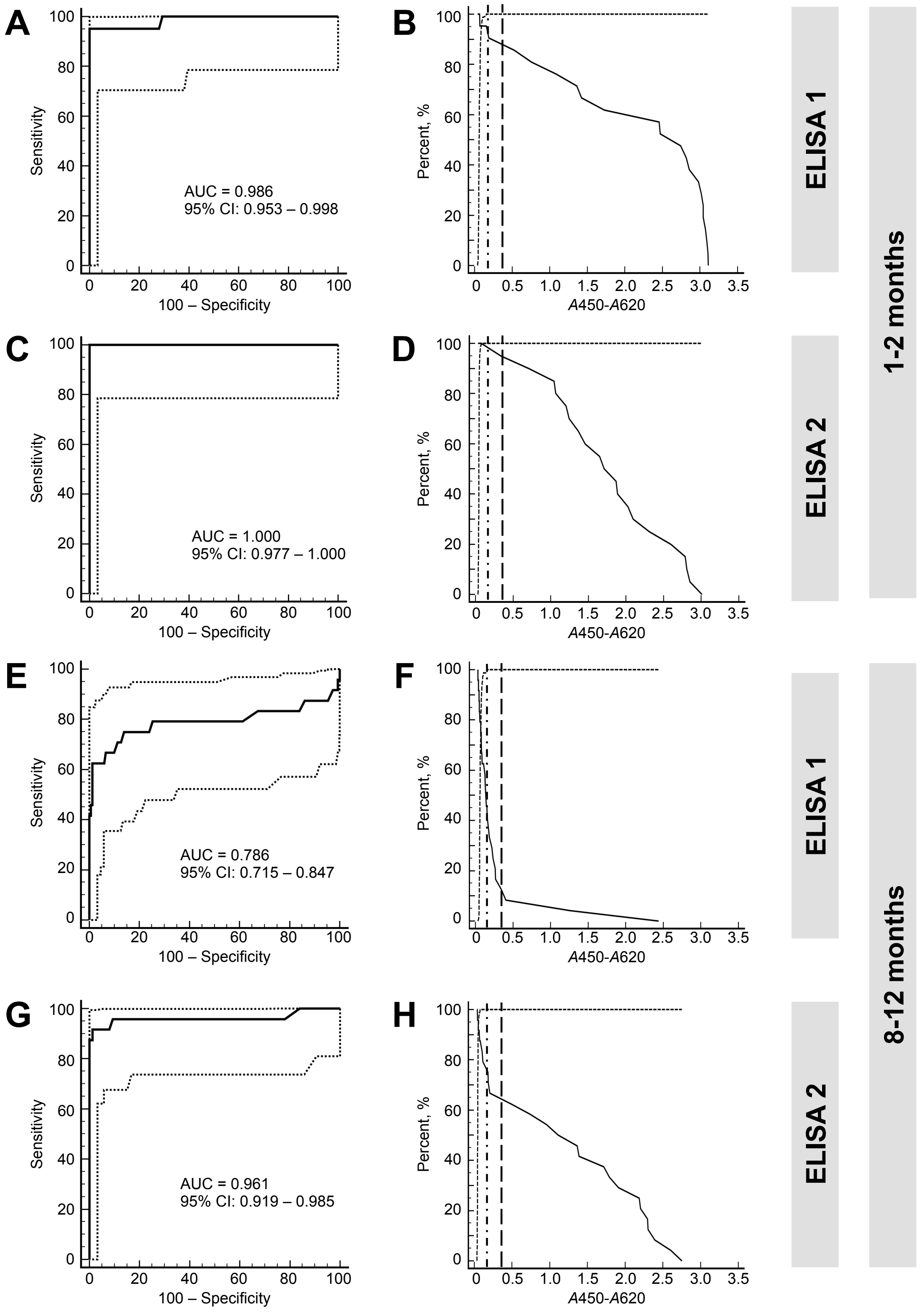

Supplemental Fig. 2: ROC analyses for ELISA 1 (**(A)**, **(B)**, **(E)**, **(F)**, sera 1:100, conjugate 1:20,000) and ELISA 2 (**(C)**, **(D)**, **(G)**, **(H)**, sera 1:2, conjugate 1:50,000) exemplified for COVID-19 patient samples collected 1-2 months (**(A)-(D)**, n=21) and 8-12 months (**(E)-(H)**, n=24) post onset of symptoms, respectively. Negative serum panel (collected before 2019): HD (healthy donors Europe, n=139). AUC: area under curve; gray line: 95% CI. (B), (D), (F), and (H): solid line: sensitivity (%), dotted line: specificity (%), dashed line: A_{cutoff} = 0.2 and = 0.4, respectively.

Supplemental Figure 3

A B

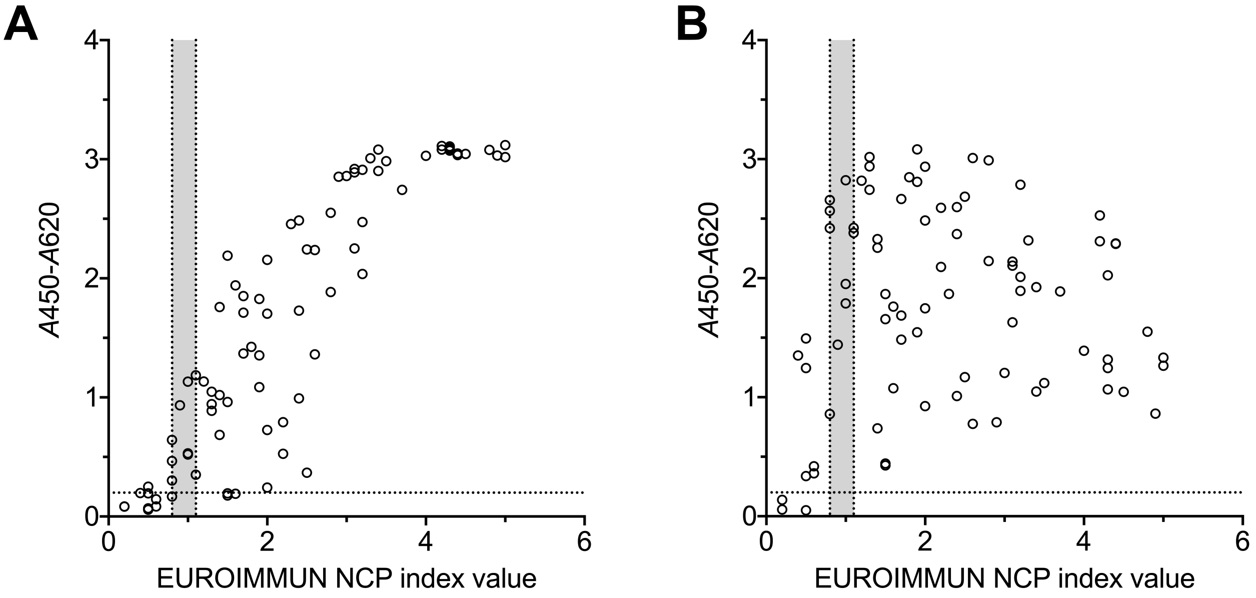

C

EUROIMMUN NCP IgG

ELISA 1 (1:100)		neg/bl	pos	Σ
	neg	9	3	12
	pos	8	62	70
	Σ	17	65	82

D

EUROIMMUN NCP IgG

ELISA 2 (1:2)		neg/bl	pos	Σ
	neg	3	0	3
	pos	14	65	79
	Σ	17	65	82

Supplemental Fig. 3. Comparison to EUROIMMUN SARS-CoV-2 IgG NCP IgG ELISA. 82 SARS-CoV-2 IgG IIFT positive serum samples originating from 25 patients (dpo range: 10-178) were analyzed with the EUROIMMUN SARS-CoV-2 NCP IgG ELISA. **(A, C)** Comparison to SARS-CoV-2 FcγR IgG ELISA 1 results. **(B, D)** Comparison to SARS-CoV-2 FcγR IgG ELISA 2 results; bl: borderline. Dashed line indicates A_{cutoff} = 0.2; grey shaded area indicates EUROIMMUN assay borderline index values.

Supplemental Figure 4

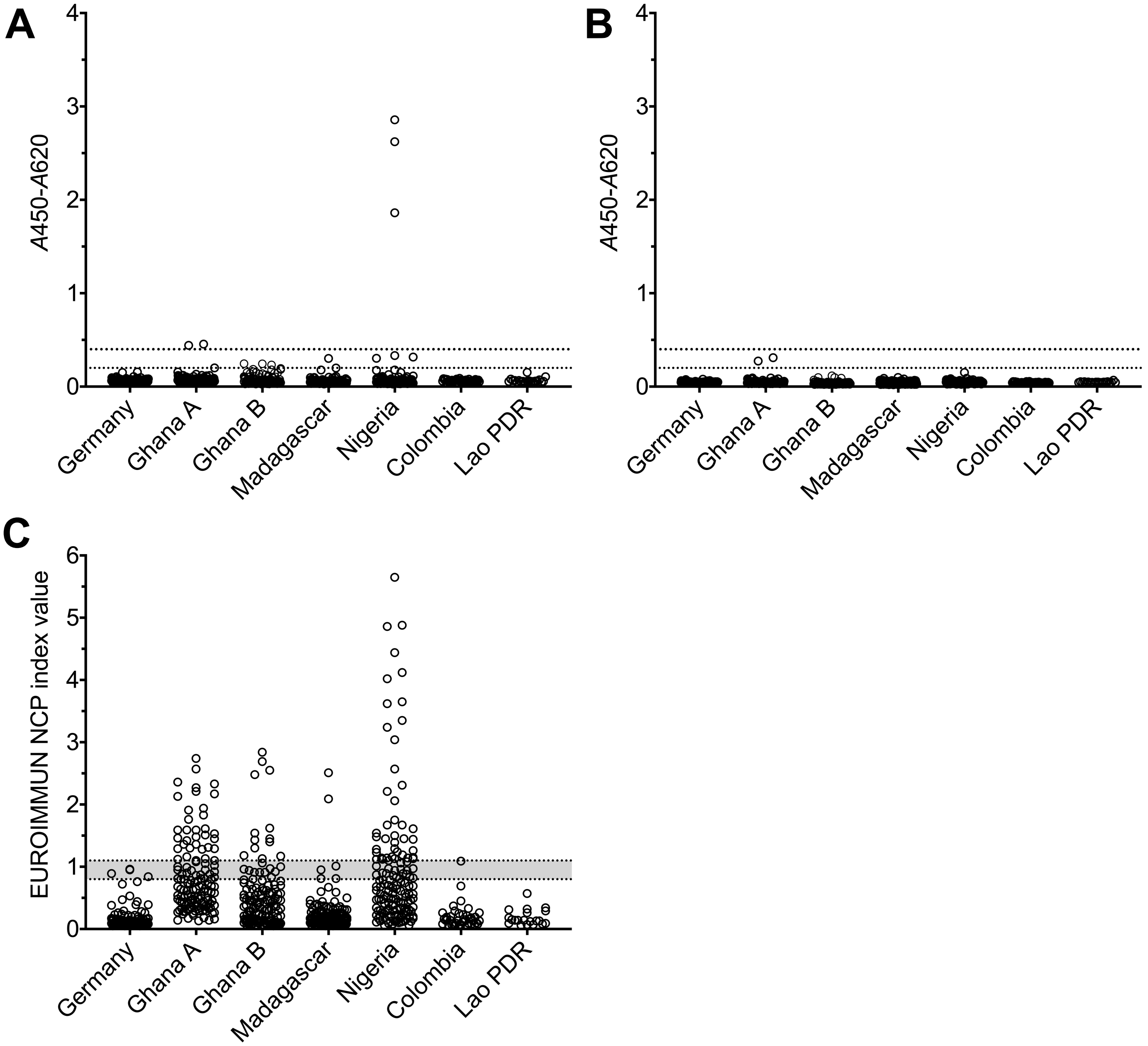

Supplemental Fig. 4. Specificity of SARS-CoV-2 IgG ELISAs. Serum/plasma panel collected from symptom-free donors before the SARS-CoV-2 pandemic in Europe (Germany: n=139), Africa (Ghana A: n=131; Ghana B: n=145; Madagascar: n=166; Nigeria: n=149), South America (Colombia: n=40), and Asia (Lao PDR: n=20) were analyzed with the SARS-CoV-2 IgG FcγR ELISAs 1 **(A)** and 2 **(B)** as well as with the EUROIMMUN SARS-CoV-2 NCP IgG ELISA **(C)**. Dashed line in (A) and (B): A_{cutoff} = 0.2 and = 0.4, respectively. Grey shaded area in (C): index values classified as "borderline" according to the manufacturer's instructions.

Supplemental Figure 5

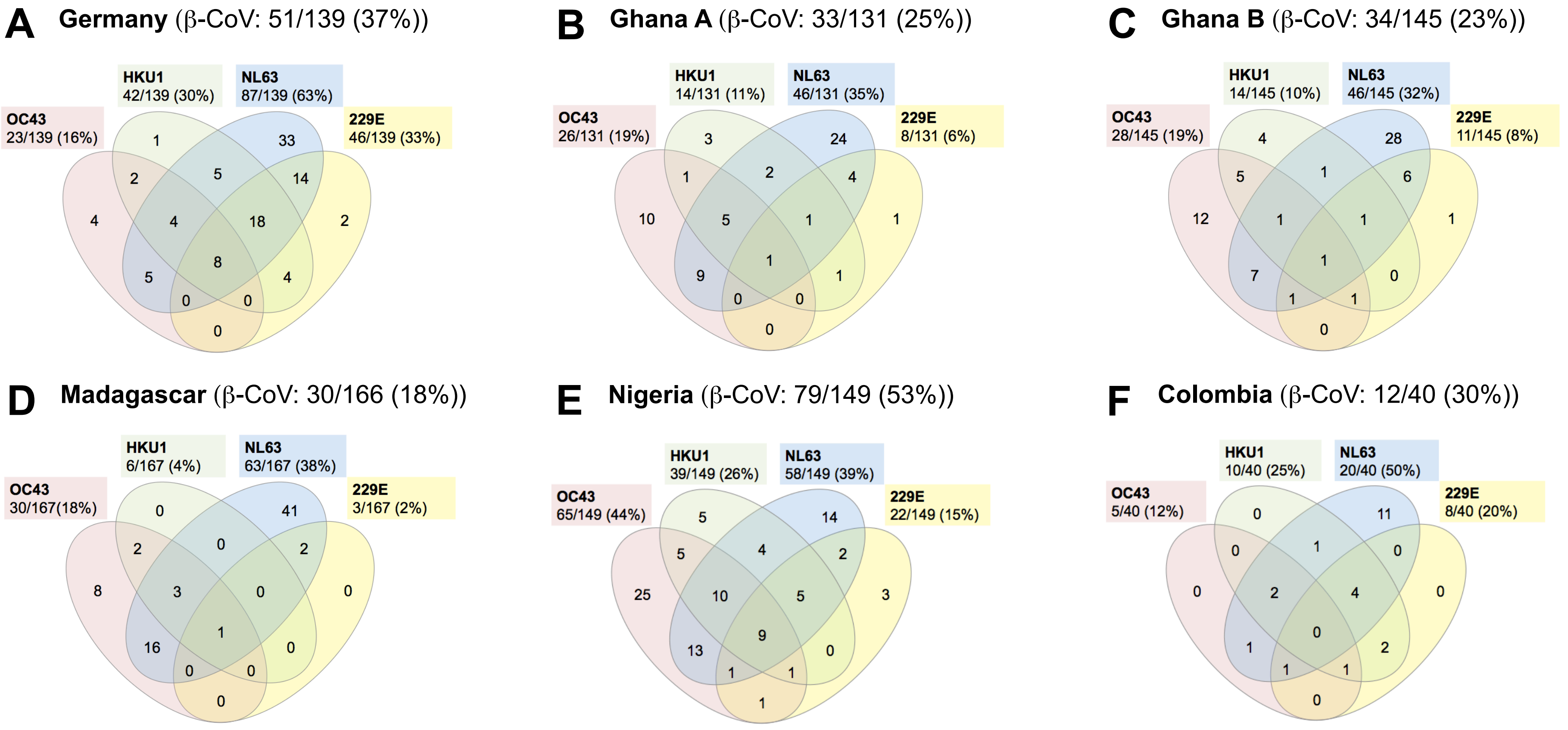

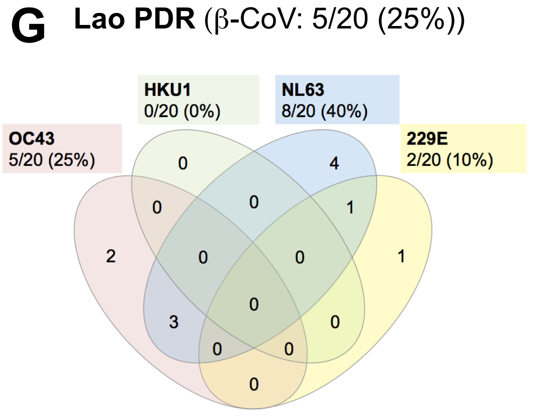

Supplemental Fig. 5. Prevalence of IgG antibodies interacting with common cold CoV NCPs in pre-pandemic negative control serum/plasma panels. Antibodies were detected in serum panels from **(A)** Germany (n=139), **(B, C)** Ghana (n_A=131, n_B=145) **(D)** Madagascar (n=166), **(E)** Nigeria (n=149), **(F)** Colombia (n=40), and **(G)** Lao PDR (n=20) using an IgG FcγR ELISA protocol employing recombinant, truncated NCPs of OC43, HKU1, NL63, or 229E as antigen. In brackets following the country's name: fraction of samples positive for IgG antibodies interacting with one or both of the two common cold β-CoVs (OC43 and/or HKU1); boxes "OC43"/"HKU1"/"NL63"/"229E": fraction of samples positive for anti-OC43/HKU1/NL63/229E IgG antibodies, respectively. Venn diagrams visualize numbers of samples testing positive in the respective common cold CoV IgG FcγR ELISAs, e.g. 18 of the 139 German samples tested positive in the HKU1, NL63, and 229E ELISA, but negative in the OC43 ELISA.
