# Supplementary material for: Fcγ-Receptor-Based Enzyme-Linked Immunosorbent Assays for Sensitive, Specific, and Persistent Detection of Anti-SARS-CoV-2 Nucleocapsid Protein IgG Antibodies in Human Sera
Source: J Clin Microbiol. 2022 May 16;60(6):e00075-22. doi: 10.1128/jcm.00075-22 (PMC9199419; doi:10.1128/jcm.00075-22)
Supplement: Supplemental file 2 — Tables S1 to S3. Download jcm.00075-22-s0002.pdf, PDF file, 0.2 MB [file jcm.00075-22-s0002.pdf]

recombinant protein	accession no. full length NCP	calculated MW (# amino acids)	oligonucleotides used for cloning	remarks
**SARS-CoV-2 NCPΔ246**	QHW06046.1	with tag: 47.3 kDa (413 aa)<br>without tag: 19.5 kDa (175 aa)	5’ – AAGTTCTGTTTCAGGGCCCG*ACTAAGAAATCTGCTGCTGAGGC* – 3’<br>5’ – CTGGTCTAGAAAGCTT*TTAGGCCTGAGTTGAGTCAGCAC* – 3’	
**SARS-CoV-1 NCPΔ247**	AAP33707.1	with tag: 47.5 kDa (415 aa)<br>without tag: 19.7 (177 aa)	5’ – AAGTTCTGTTTCAGGGCCCG*ACTAAGAAATCTGCTGCTGAGGCATC* – 3’<br>5’ – CTGGTCTAGAAAGCTT*TTATGCCTGAGTTGAATCAGCAGAAG* – 3’	
**MERS NCPΔ238**	AGN70936.1	with tag: 47.8 kDa (415 aa)<br>without tag: 20.0 kDa (177 aa)	5’ – AAGTTCTGTTTCAGGGCCCG*ACCAAGAAGGACGCCGCGGC* – 3’<br>5’ – CTGGTCTAGAAAGCTT*TTAGTCCGTATTCACATCAATCATCGG* – 3’	codon optimized (*E. coli*)
**HKU1 NCPΔ256**	ARU07581.1, S290F	with tag: 48.6 kDa (425 aa)<br>without tag: 20.8 kDa (187 aa)	5’ – AAGTTCTGTTTCAGGGCCCG*GCGAAGGAGATCCGCCACAAAATC* – 3’<br>5’ – CTGGTCTAGAAAGCTT*TTAGGCCACGGAATCTTCCACG* – 3’	codon optimized (*E. coli*)
**OC43 NCPΔ258**	AMM42450.1, A49T	with tag: 49.2 kDa (430 aa)<br>without tag: 21.4 kDa (192 aa)	5’ – AAGTTCTGTTTCAGGGCCCG*GCCAAAGAAGTCAGACAGAAAATTTTG* – 3’<br>5’ – CTGGTCTAGAAAGCTT*TTATATTTCTGAGGTGTCTTCAGTAAAGG* – 3’	
**NL63 NCPΔ222**	ABI20791.1	with tag: 45.3 kDa (395 aa)<br>without tag: 17.5 kDa (157 aa)	5’ – AAGTTCTGTTTCAGGGCCCG*CCGCGCGCGGATAAAC* – 3’<br>5’ – CTGGTCTAGAAAGCTT*TTAATGCAGCACTTCATTAACGATCTC* – 3’	codon optimized (*E. coli*)
**229E NCPΔ235**	NP_073556.1	with tag: 45.1 kDa (394 aa)<br>without tag: 17.3 kDa (156 aa)	5’ – AAGTTCTGTTTCAGGGCCCG*GAGACGAAAGAGCAGAAGCATG* – 3’<br>5’ – CTGGTCTAGAAAGCTT*TTAATTAACTTCATCGATGATATCGGTTTC* – 3’	codon optimized (*E. coli*)

Supplemental Table 1. Survey of recombinant CoV NCP proteins. MW: molecular weight; aa: amino acids; *italics*: CoV NCP coding sequence; underlined: 15 bp vector homology sequence for in-fusion cloning.

Serum panel	Germany	Ghana A	Ghana B	Madagascar	Nigeria	Colombia	Lao PDR
**Number**	139	131	145	166	149	40	20
**Sampling year**	2004 - 2015	1999	2014 - 2015	2010	2018	2014	2014
**Sample type**	Serum	Serum	Serum	Plasma	Serum	Serum	Serum
**Age (median, IQR)**	39 (28-48)[§]	22 (16-44)*	6 (3-7)	24 (20-30)**	42 (30-58)[#]	23 (21-25)	48 (45-53)
**Sex m/f (n (%))**	72/34 (68/32)[§]	70/61 (53/47)	69/76 (48/52)	166/0 (100/0)[$]	79/70 (53/47)	17/23 (43/57)	3/17 (15/85)
**Sampling site**	Hamburg	Villages in the central region of Ghana	Agogo	Manakara, Marofarihy, Tsiroanomandidy, Mananjary	Irrua	Valledupar	Savannakhet

Supplemental Table 2. A priori SARS-CoV-2 IgG negative serum/plasma panels. All samples were collected from symptom-free donors prior to the COVID-19 pandemic. IQR: interquartile range (25%-75%); m: male; f: female; n: number.

[§] n=106 (age and sex information not available for 33 donors (all 33: age ≥ 18 years))

* n= 88 (age information not available for 43 donors (all 43: age ≥ 18 years))

** n=164 (age information not available for two donors)

[$] all donors: pregnant women

[#] n=148 (age information not available for one donor)

(A) ELISA 1 (sera 1:100, conjugate 1:20,000)

	day 1			day 2			day 3						
**sample**	**mean**	**sd**	**CV_{intra}**	**mean**	**sd**	**CV_{intra}**	**mean**	**sd**	**CV_{intra}**	**$CV_{intra, av}$**	**$mean_{av}$**	**sd of $mean_{av}$**	**CV_{inter}**
pos1a	0.919	0.035	3.8	0.837	0.009	1.0	0.865	0.033	3.8	2.9	0.874	0.042	4.8
pos2a	0.492	0.018	3.6	0.528	0.027	5.2	0.498	0.020	4.1	4.3	0.506	0.020	3.9
pos3a	0.901	0.078	8.6	0.935	0.066	7.1	0.964	0.036	3.7	6.5	0.933	0.032	3.4
pos4a	2.053	0.030	1.5	1.987	0.046	2.3	2.042	0.062	3.0	2.3	2.028	0.035	1.7
neg1a	0.057	0.005	9.1	0.053	0.006	10.8	0.048	0.003	5.3	8.4	0.052	0.005	8.9
neg2a	0.053	0.001	1.9	0.053	0.010	19.2	0.047	0.003	5.6	8.9	0.051	0.004	7.0
neg3a	0.066	0.001	0.9	0.063	0.001	1.8	0.061	0.001	0.9	1.2	0.063	0.002	3.4
neg4a	0.049	0.001	1.2	0.046	0.002	3.8	0.048	0.002	4.4	3.1	0.047	0.001	2.8
										**$mean_{pos}$: 4.0**			**$mean_{pos}$: 3.4**
										**$mean_{neg}$: 5.4**			**$mean_{neg}$: 5.5**

(B) ELISA 2 (sera 1:2, conjugate 1:50,000)

	day 1			day 2			day 3						
**sample**	**mean**	**sd**	**CV_{intra}**	**mean**	**sd**	**CV_{intra}**	**mean**	**sd**	**CV_{intra}**	**$CV_{intra, av}$**	**$mean_{av}$**	**sd of $mean_{av}$**	**CV_{inter}**
pos1b	0.620	0.007	1.2	0.529	0.022	4.1	0.505	0.018	3.6	2.9	0.551	0.061	11.0
pos2b	1.065	0.034	3.2	0.981	0.036	3.7	0.941	0.065	6.9	4.6	0.996	0.064	6.4
pos3b	1.832	0.059	3.2	1.736	0.033	1.9	1.711	0.058	3.4	2.8	1.760	0.064	3.6
pos4b	2.564	0.071	2.8	2.428	0.066	2.7	2.443	0.160	6.5	4.0	2.478	0.075	3.0
neg1b	0.061	0.006	10.0	0.052	0.001	2.2	0.040	0.001	2.5	4.9	0.051	0.011	20.9
neg2b	0.058	0.000	0.0	0.051	0.004	7.8	0.041	0.001	2.8	3.5	0.050	0.008	16.7
neg3b	0.059	0.001	1.0	0.051	0.002	4.1	0.040	0.001	1.4	2.2	0.050	0.009	18.4
neg4b	0.053	0.003	5.8	0.048	0.002	3.2	0.039	0.002	4.0	4.3	0.047	0.007	15.4
										**$mean_{pos}$: 3.6**			**$mean_{pos}$: 6.0**
										**$mean_{neg}$: 3.7**			**$mean_{neg}$: 17.9**

Supplemental Table 3. Reproducibility of SARS-CoV-2 IgG FcγR ELISAs. *A*450-*A*620 values were determined in triplicate for four anti-SARS-CoV-2-NCP IgG positive serum samples and four anti-SARS-CoV-2-NCP IgG negative serum samples on three different days with **(A)** SARS-CoV-2 IgG FcγR ELISA 1 (sera 1:100, conjugate 1:20,000) and **(B)** SARS-CoV-2 IgG FcγR ELISA 2 (sera 1:2, conjugate 1:50,000). Sd: standard deviation, av: average; CV_{intra}: intraassay variation, CV_{inter}: interassay variation.
