# Supplementary material for: Fcγ-Receptor-Based Enzyme-Linked Immunosorbent Assays for Sensitive, Specific, and Persistent Detection of Anti-SARS-CoV-2 Nucleocapsid Protein IgG Antibodies in Human Sera
Source: J Clin Microbiol. 2022 May 16;60(6):e00075-22. doi: 10.1128/jcm.00075-22 (PMC9199419; doi:10.1128/jcm.00075-22)
Supplement: Supplemental file 3 — STARD checklist and flowchart. Download jcm.00075-22-s0003.pdf, PDF file, 0.1 MB [file jcm.00075-22-s0003.pdf]

STARD Checklist

Section and Topic	No.	Item	Reported in section/paragraph
**TITLE or ABSTRACT**	1	Identification as a study of diagnostic accuracy using at least one measure of accuracy (such as sensitivity, specificity, predictive values, or AUC)	Title, Abstract
**ABSTRACT**	2	Structured summary of study design, methods, results, and conclusions (for specific guidance, see STARD for Abstracts)	Abstract
**INTRODUCTION**	3	Scientific and clinical background, including the intended use and clinical role of the index test	Introduction
	4	Study objectives and hypotheses	
**METHODS**			
Study design	5	Whether data collection was planned before the index test and reference standard were performed (prospective study) or after (retrospective study)	Section "Material and Methods", paragraph "Human sera"; Supplemental Table 2
Participants	6	Eligibility criteria	
	7	On what basis potentially eligible participants were identified (such as symptoms, results from previous tests, inclusion in registry)	
	8	Where and when potentially eligible participants were identified (setting, location and dates)	
	9	Whether participants formed a consecutive, random or convenience series	
Test methods	10a	Index test, in sufficient detail to allow replication	Section "Material and Methods", paragraph "SARS-CoV-2 IgG ELISAs"
	10b	Reference standard, in sufficient detail to allow replication	Section "Material and Methods", paragraphs "SARS-CoV-2 IgG IIFT" and "SARS-CoV-2 IgG ELISAs"
	11	Rationale for choosing the reference standard (if alternatives exist)	
	12a	Definition of and rationale for test positivity cut-offs or result categories of the index test, distinguishing pre-specified from exploratory	Section "Material and Methods", paragraph "Data analysis" ; Section "Results",

			paragraph “Diagnostic sensitivity”
	12b	Definition of and rationale for test positivity cut-offs or result categories of the reference standard, distinguishing pre-specified from exploratory	Section “Material and Methods”, paragraph “SARS-CoV-2 IgG ELISAs”
	13a	Whether clinical information and reference standard results were available to the performers/readers of the index test	Section “Material and Methods”, paragraph “Human sera”
	13b	Whether clinical information and index test results were available to the assessors of the reference standard	
Analysis	14	Methods for estimating or comparing measures of diagnostic accuracy	Section “Material and Methods”, paragraph “Data analysis”
	15	How indeterminate index test or reference standard results were handled	Legend to Supplemental Figure 4
	16	How missing data on the index test and reference standard were handled	Legend to Supplemental Figure 3
	17	Any analyses of variability in diagnostic accuracy, distinguishing pre-specified from exploratory	not applicable
	18	Intended sample size and how it was determined	
**RESULTS**			
Participants	19	Flow of participants, using a diagram	STARD Flowchart
	20	Baseline demographic and clinical characteristics of participants	Section “Material and Methods”, paragraph “Human sera”, Supplemental Table 2
	21a	Distribution of severity of disease in those with the target condition	
	21b	Distribution of alternative diagnoses in those without the target condition	
	22	Time interval and any clinical interventions between index test and reference standard	not applicable
Test results	23	Cross tabulation of the index test results (or their distribution) by the results of the reference standard	Figure 4, Table 1, Table 2, Supplemental Figure 3, Supplemental Figure 4

	24	Estimates of diagnostic accuracy and their precision (such as 95% confidence intervals)	Section “Results”, paragraph “Diagnostic sensitivity”, Figure 3, Table 1
	25	Any adverse events from performing the index test or the reference standard	not applicable
**DISCUSSION**	26	Study limitations, including sources of potential bias, statistical uncertainty, and generalisability	Section “Discussion”, paragraph “Caveats and limitations”
	27	Implications for practice, including the intended use and clinical role of the index test	
**OTHER INFORMATION**	28	Registration number and name of registry	HRECs (Human research Ethics Committees): Section “Material and Methods”, paragraph “Human sera”
	29	Where the full study protocol can be accessed	not applicable
	30	Sources of funding and other support; role of funders	Section “Authors’ Disclosures, Potential Conflicts of Interest”

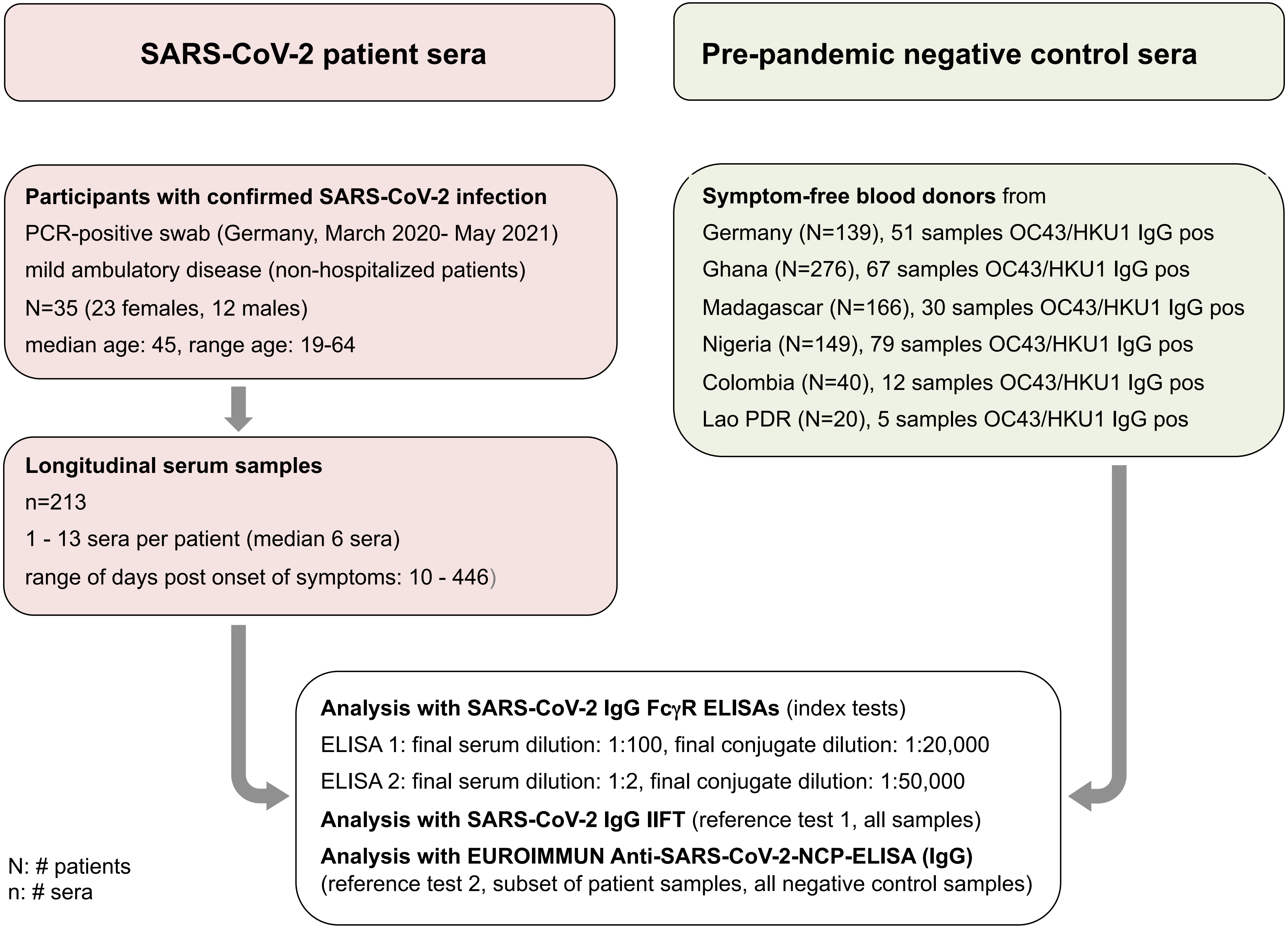
SARS-CoV-2 patient sera
Pre-pandemic negative control sera
Participants with confirmed SARS-CoV-2 infection
PCR-positive swab (Germany, March 2020- May 2021)
mild ambulatory disease (non-hospitalized patients)
N=35 (23 females, 12 males)
median age: 45, range age: 19-64
Symptom-free blood donors from
Germany (N=139), 51 samples OC43/HKU1 IgG pos
Ghana (N=276), 67 samples OC43/HKU1 IgG pos
Madagascar (N=166), 30 samples OC43/HKU1 IgG pos
Nigeria (N=149), 79 samples OC43/HKU1 IgG pos
Colombia (N=40), 12 samples OC43/HKU1 IgG pos
Lao PDR (N=20), 5 samples OC43/HKU1 IgG pos
Longitudinal serum samples
n=213
1 - 13 sera per patient (median 6 sera)
range of days post onset of symptoms: 10 - 446)
Analysis with SARS-CoV-2 IgG FcγR ELISAs (index tests)
ELISA 1: final serum dilution: 1:100, final conjugate dilution: 1:20,000
ELISA 2: final serum dilution: 1:2, final conjugate dilution: 1:50,000
Analysis with SARS-CoV-2 IgG IIFT (reference test 1, all samples)
Analysis with EUROIMMUN Anti-SARS-CoV-2-NCP-ELISA (IgG)
(reference test 2, subset of patient samples, all negative control samples)
N: # patients
n: # sera
